# Scoping review about the professional integration of internationally educated health professionals

**DOI:** 10.1186/s12960-016-0135-6

**Published:** 2016-06-17

**Authors:** Christine L. Covell, Elena Neiterman, Ivy Lynn Bourgeault

**Affiliations:** Faculty of Nursing, University of Alberta, 5-301, ECHA, 11405-87 Avenue, Edmonton, T6G 1C9 Alberta Canada; School of Public Health and Health Systems, University of Waterloo, Waterloo, Ontario Canada; Telfer School of Management, University of Ottawa, Ottawa, Ontario Canada

**Keywords:** Internationally educated health professionals, Scoping review, Professional integration, Canada

## Abstract

**Background:**

Over the last decade, Canada has been one of the top destination countries for internationally educated health professionals (IEHPs). After arrival, many struggle to professionally recertify and secure employment in their field. Considerable funding has been allocated to the development of new policies and programs to facilitate IEHPs’ professional integration. Literature about the professional integration process and the available policies and programs is abundant, not synthesized and dispersed among a wide range of health professions and the academic and grey literature. This, in combination with the sustained policy relevance, contributed to the timeliness and necessity for conducting this scoping review.

**Methods:**

We used an updated version of Arskey and O’Malley’s six-stage scoping review framework to summarize the amount, types, sources and distribution of the literature. Findings were summarized numerically and thematically. The themes included pre-immigration activities and programs, early arrival activities and programs, professional recertification and workplace integration.

**Results:**

Four hundred and seven published sources from 2000–2012 were retained for data charting and extraction. Most focused on international medical graduates or internationally educated nurses. IEHPs from the allied health professions were underrepresented. Methodologically, about one quarter of the papers are empirical studies with the next largest category being reports from professional certification bodies and educational institutions. The overarching concern is with workplace integration, professional recognition and bridging programs. Nursing dominates the literature about pre-immigration activities and programs whereas the literature about early arrival activities and programs, professional recertification and workplace integration is dominated by medicine. Although the literature does contain some information for IEHPs in the allied health professions, the thematic analysis did not identify a clear trend. A notable increase in the number of publications was present.

**Conclusions:**

The literature about IEHPs’ professional integration in Canada is abundant. This reflects the sustained policy relevance of the recruitment, recognition and professional integration for IEHPs in Canada. This demonstrates that Canada provides an excellent case for this review from which the findings may have international significance. Nevertheless, little information is available about the effectiveness of the policies and programs available to facilitate IEHP integration, an area that requires further consideration.

**Electronic supplementary material:**

The online version of this article (doi:10.1186/s12960-016-0135-6) contains supplementary material, which is available to authorized users.

## Background

In this paper, we review what is known about the professional integration of internationally educated health professionals (IEHPs) in Canada. Canada is one of a handful of so-called destination countries for migrating health workers. Like the United States of America, the United Kingdom and Australia, IEHPs have always been an integral part of the Canadian health care workforce, with 25 % of physicians and 8 % of nurses being internationally trained [1, 2]. There are also growing concerns about a number of immigrants with health professional training who are not working in their profession. Canada provides a unique case for examining the professional integration of IEHPs due to the social and financial commitment made to immigrants in general and to IEHPs in particular. It represents, perhaps, the ‘best case scenario’ for IEHP integration; hence, the insights garnered from a review of this case are of international significance. The federated nature of the Canadian health system within which IEHPs are seeking integration also represents an internally comparative perspective across provincial and territorial jurisdictions.

Considerable emphasis both nationally and internationally has been given to focusing on IEHPs as a potential solution for addressing the shortages of health human resources. Although IEHPs have always migrated, in the last decade, high numbers of IEHPs, especially from developing countries, have migrated to Canada independently or through private or provincial recruitment initiatives. After arrival, IEHPs often struggle to become recertified and employed within their profession. As a result, several million dollars have been devoted to simplifying the recognition process and facilitating the professional integration of IEHPs. These investments have led to the creation of new policies, programs and promising practices to support IEHPs in Canada. While there has been a significant amount of literature published about IEHPs, our knowledge in this area is affected as the information is not synthesized but rather dispersed between the academic and grey literature [3] and covers a wide range of health professions. No knowledge synthesis in this critically important area of health human resources and policy existed. Therefore, the growing body of literature as well as the sustained policy relevance of the recognition and integration of IEHPs in Canada demonstrates the timeliness and necessity for conducting this scoping review.

The aim of this scoping review is to map key themes in the literature on the professional integration of IEHPs in Canada. The objectives were to summarize the amount, types, sources and distribution and identify key themes in the literature. The gaps in the literature are discussed.

## Methods

The project began by assembling a team of five researchers who were experts in scoping review methodology and in the content area of IEHPs. We also formed an Advisory Committee of 15 stakeholders—representatives from academia, federal and provincial and territorial government organizations, and professional regulatory colleges and associations that work with or on behalf of IEHPs. The Advisory Committee provided the team with expert advice throughout the project.

A six-stage methodological framework for scoping reviews guided the approach [4–6]. The methodological framework was used to standardize and clarify the procedures we used at each stage of the review. The process employed to conduct the scoping review is presented below with a description of each stage of the methodological framework.

**Fig. 1:**
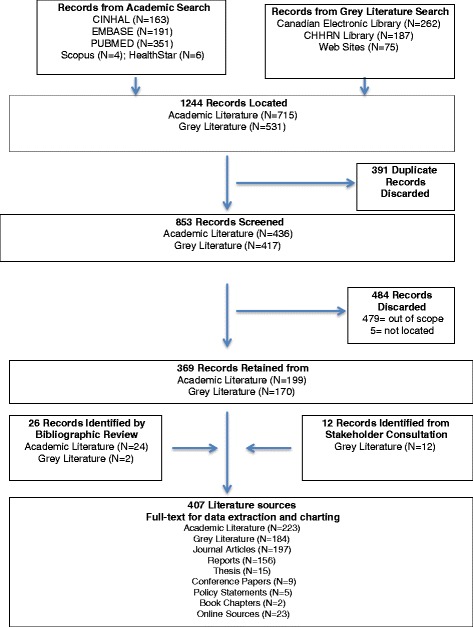
Academic and grey literature search
- Stage 4: extracting and charting the data
  To ensure standardization of data extraction and charting across the team [5], we developed a charting tool in Microsoft Excel. The categories for data extraction reflected our research questions: type of paper (journal articles, reports, theses, conference papers, book chapters, policy statements, online sources); health profession (internationally educated nurses (IENs); internationally educated midwives (IEMs); international medical graduates (IMGs); internationally educated health professional-no specific profession (IEHPs); internationally educated occupational therapists (IEOTs); internationally educated respiratory therapists (IERTs); internationally trained dentists (ITD); internationally trained pharmacists (ITPs); internationally educated medical radiation technologists (IEMRTs); internationally educated physical therapists (IEPTs); internationally educated medical laboratory technologists (IEMLTs)); research method (qualitative, quantitative, mixed-method) where applicable; geographic location (pan-Canadian, province or territory); and themes.
  The themes were developed after each team member carefully read and reread the literature. An a priori template of major themes was developed to reflect the stages of IEHPs’ professional integration: pre-immigration activities and programs, early arrival activities and programs, professional recertification, alternate paths to professional integration and workplace integration. As shown in Table 1, standard definitions were developed for each major theme. Minor themes were developed inductively and used to organize the information within each major theme. To ensure that the data extraction process was reliable, in that it was consistent with the research questions, each team member used the charting tool to independently extract data from 10 papers and the results were compared [5]. Discrepancies in the coding were discussed among the team, and the tool was refined before proceeding with data extraction in a consistent and standardized manner.

- Stage 1: identifying the research questions
  A scoping review is intended to summarize a large amount of literature on a topic. The research questions for our review were intentionally broad [5]: What is the scope of the literature about IEHPs in Canada including the amount, type, sources, distribution and focus of the conceptual and empirical literature? What are the gaps in the literature?
- Stage 2: identifying the academic and grey literature
  The inclusion criteria for identifying the literature were the publication: topic was IEHPs in Canada, was issued from 2000 to 2012 and was in the English or French language. Opinion pieces, press releases, symposia proceedings and letters to the editor were excluded.
  Multiple strategies were used to locate both the academic and the grey literature. The search strategy developed by a research librarian included keywords (used alone and in combination) of as follows: “foreign professional personnel” “internationally educated,” “internationally trained” “international graduates” “foreign-trained,” “foreign educated” “foreign graduates” “health professional” “nurses” “physicians” “dentists” “pharmacists” ‘health occupations” “immigrant,” “migrant” “integration” “licensure” “job satisfaction” “Canada ” “Nova Scotia’ “Newfoundland” “Prince Edward Island” “New Brunswick” “Quebec” “ Ontario” “Manitoba” “Saskatchewan” “Alberta” “British Columbia” “North West Territories” “Yukon” and Nunavut”. The academic literature searched through the electronic databases CINAHL, Embase, PubMed, Healthstar and Scopus produced 715 records.
  The grey literature was searched through the Canadian Electronic Library, the Canadian Health Human Resources Network Library and the websites of federal, provincial and territorial governments and professional and immigrant associations by using the same keywords (alone and in combination). The grey literature search produced 527 records.
- Stage 3: selecting the literature
  A systematic process was used to select the literature for the scoping review. First, the results of the searches were imported into a reference-management program. Of the 1244 records, 391 duplicate records were identified and discarded. Two members of the team then screened the abstracts of the remaining 853 records to determine their relevance to the review’s purpose and research questions. An additional 479 records were deemed out of the scope of the review and subsequently discarded. Five papers could not be located. Resulting in the retention of 369 records. Bibliographic reviews of the remaining papers produced an additional 26 papers. Stakeholders from the Advisory Committee identified 12 other literature sources. Ultimately, a total of 407 papers were brought forward for data extraction and charting (See Additional file 1: Table S1). Figure 1 summarizes the literature search and selection.

**Table 1:** Narrative themes

Major theme	Definition	Minor themes
Pre-immigration activities and programs	The activities IEHPs undertake prior to migration and upon arrival to prepare for practicing their profession in Canada.	▪ Push and pull factors ▪ International recruitment ▪ Pre-immigration testing or verification
Early arrival activities and programs	The activities IEHPs undertake upon arrival and the programs available to prepare them for practicing their profession in Canada.	▪ Arrival and system navigation ▪ Early arrival programs ▪ Health profession specific associations
Credential recognition and professional recertification	The process IEHPs engage in to meet the requirements for registration with a professional regulatory college in Canada.	▪ Barriers and facilitators to IEHP professional recertification ▪ Stakeholders’ recognition of the barriers to IEHP professional recertification ▪ Programs and policies to facilitate IEHP professional recertification
Bridging and residency training programs	Programs that provide educational, mentorship or clerkship opportunities to facilitate professional integration of IEHPs.	▪ Bridging programs ▪ Residency programs ▪ Direct to work bridging programs ▪ Facilitators and barriers of bridging and residency programs
Alternate paths to professional integration	Examples of IEHPs pursuing integration via other professional roles or jobs.	▪ Alternate path for IMGS ▪ Alternate paths for IENs ▪ Alternate paths for other IEHPs
Workplace integration	When IEHPs become members of a workgroup within an organization where they can use their professional knowledge and expertise.	▪ Practice profile of IEHPs ▪ Workplace discrimination ▪ Other facilitators and barriers ▪ Role of employers
- Stage 5: collating, summarizing and reporting the results
  The extracted data was collated into numerical and qualitative thematic summaries. To address the research questions, we used frequencies to report the numerical data. The qualitative data were summarizing into narrative syntheses. The findings were analyzed in relation to the purpose of the scoping review and the research questions. Gaps in the literature were identified. We did not appraise the quality of the literature, as is indicated for scoping reviews [4, 7].
- Stage 6: consultation
  The Advisory Committee created at the onset of the project was consulted via in-person meetings and teleconferences at several stages during the review. During the first stage, the Advisory Committee provided feedback on the research questions. In the second stage, they made suggestions for additional literature we may have missed. In stage four, they provided feedback regarding the themes used for extraction to determine if they are relevant for decision-making and policy development. At stage five, they provided feedback on the findings at an in-person workshop.

## Results

General information about the types and sources of literature identified during the charting process is presented below, followed by the numerical and qualitative thematic analyses. The gaps in literature are highlighted within each qualitative theme.

### Types and sources of literature

Of the 407 sources, 197 (48 %) are journal articles and 156 (38 %) are reports issued by government agencies, professional associations, non-governmental organizations or academia. The remaining 54 (13 %) sources are theses (*n* = 15), conference papers (*n* = 9), policy statements (*n* = 5), book chapters (*n* = 2) and online sources such as webpages, electronic articles, blogs and data sets (*n* = 23).

Almost half of the literature used an empirical method (*n* = 189; 46 %). Nearly one half (48 %, *n* = 91) used quantitative, 15 % (*n* = 62) qualitative and 19 % (*n* = 33) mixed methods designs.

**Fig. 2:**
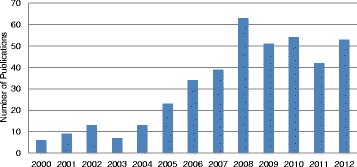
Number of sources by year of publication (*N* = 407)

The geographical distribution of the publications was primarily pan-Canadian (*n* = 233; 57 %). Nearly one fifth (*n* = 77; 19 %) of the literature was authored by individuals located in the Province of Ontario. The literature was mostly focused on IMGs (*n* = 196, 40 %) and IENs (*n* = 126, 25 %). The remaining sources concentrate on allied health professionals (*n* = 165; 33 %) or are about IEHPs in general and do not identify a professional group (*n* = 109; 22 %). An evident trend in an increase in the number of publications was present, with 2008 as the peak year of publication (Fig. 2).

### Numerical thematic findings

**Fig. 3:**
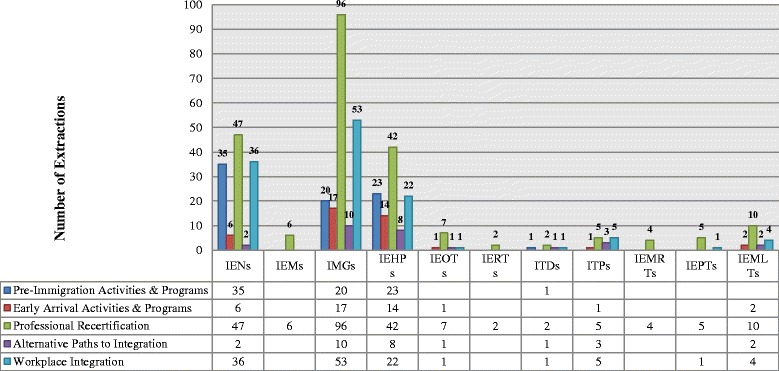
Frequency of themes by profession (*N* = 496)

As Fig. 3 demonstrates, the numerical analysis of the themes resulted in 496 extractions. The findings reveal that the literature is primarily about professional recertification (*n* = 226, 42 %). The workplace integration (*n* = 123; 25 %) and pre-immigration activities and programs themes were moderately represented (*n* = 79; 16 %). Considerably less information is available about early arrival activities and programs (*n* = 41, 8 %) and alternate paths to professional integration (*n* = 27; 5 %). For the thematic coverage per paper, see Additional file 2: Table S2.

Greater than one fifth (*n* = 109, 22 %) of the extractions did not identify a specific professional group and are coded as IEHPs. The frequency of the remaining thematic extractions varied by profession. Nursing dominates the literature about pre-immigration activities and programs (*n* = 35; 44 %). The literature about early arrival activities and programs (*n* = 17, 41 %) and professional recertification (*n* = 96; 42 %) and workplace integration (*n* = 53, 11 %) is dominated by medicine. Although the literature does contain some information for IEHPs in the allied health professions (*n* = 65, 13 %), the thematic analysis did not identify a clear trend.

### Qualitative thematic findings

Findings were summarized according to the five themes: pre-immigration activities and programs, early arrival activities and programs, professional recertification, alternate paths to professional integration and workplace integration. Gaps identified in the literature are presented per theme.

### Pre-immigration activities and programs

The pre-immigration activities and programs literature discusses the activities IEHPs undertake prior to migration and upon arrival to prepare for practicing their profession in Canada. The literature details how the circumstances surrounding IEHPs’ immigration influences the type of activities they undertake prior to immigration. The literature indicates that some IEHPs have significant time and ability to plan their move to Canada, and others, like refugees, move with little preparation. Of the 79 papers that cover the theme, nearly one half (*n* = 35, 44 %) were about IENs, followed by IEHPs (*n* = 23, 29 %) and IMGs (*n* = 20, 25 %).

#### Push and pull factors

Several papers explore the motivations behind IEHPs’ decisions to emigrate. Push factors include broad political, financial and social problems in their home country; professional issues such as lack of medical resources, poor working conditions or low remuneration; and personal incentives such as a poor quality of life for their families and low educational opportunities for their children [8, 9]. Many IEHPs describe a confluence of factors motivating them to emigrate and find it difficult to identify specific push factors [10]. The reasons that pull IEHPs to Canada include positive views of the Canadian society and governance, professional considerations and personal incentives [11]. An overriding motivation for selecting Canada is IEHPs’ perceptions that health care workers are in demand and they will obtain preference in the immigrant selection process [12].

#### International recruitment

The issue of international recruitment is particularly salient in the medical, nursing and pharmacy literature. Although Canada does not actively recruit health professionals at a national level, the recruitment of IEHPs for specific positions within the health care systems has been carried out by private companies, placement agencies or through focused provincial and territorial or regional health authorities. There is sizeable amount literature addressing the ethics of international recruitment. Of primary concern is the impact on “sending” countries and whether IEHPs are gainfully re-employed within the Canadian health care system. Recruitment from countries with health worker shortages is generally perceived as unethical practice [13]. Most literature does, however, recognize professionals’ right to migrate and suggests attention should be directed towards improving the factors that push professionals to emigrate rather than prevent emigration [14, 15].

#### Pre-immigration testing or verification

The literature emphasizes the importance of immigrants having access to information prior to immigrating to help them adequately plan for re-establishing practice in their professions upon arrival to Canada [16]. The literature also recommends IEHPs gather all relevant documents before emigrating, especially IEHPs from politically unstable countries or from countries that have poor record keeping [17]. Completing the credential assessment and verification process, perfecting their language skills and taking the professional examination for licensure before immigrating are also recommended [18, 19]. Yet despite some examinations being offered overseas, only small numbers of IEHPs take the examinations prior to becoming permanent residents of Canada [20].

In summary, migration is a complex and complicated process that occurs in the context of personal factors and social and structural conditions (e.g. push and pull factors, international recruitment). While it has been known for some time that this is so, we have yet to identify the most dominant or least dominant factors in the push-pull model of migration. We also have less literature about the ethics of international recruitment of allied health professionals. In the Canadian context, specifically, it is noted that IEHPs do not always fully prepare to migrate, which has not been fully explored. Some possible explanations could be the lack of information or access to exams when overseas and misinterpreting the immigration point system as a measure of their ability to become registered and employed immediately upon arrival. Lack of preparation before migrating can have a negative effect on IEHPs’ ability to acquire employment in their profession once in Canada.

### Early arrival activities and programs

The activities IEHPs undertake upon arrival and the programs available to prepare them for practicing their profession in Canada. Eight percent (*n* = 41) of the literature about IEHPs centres on the activities IEHPs undertake upon arrival and the programs available to prepare them for practicing their profession in Canada. The literature primarily focuses on the challenges IEHPs encounter and the policy solutions and support available to them upon their arrival. While this literature does speak to IMGs (*n* = 17, 41 %) and about IEHPs in general (*n* = 14, 34 %), there is an absence of literature that focuses on the early arrival activities and programs for IEHPs in the allied health professions.

#### Challenges experience by IEHPs

The literature recognizes the greatest barrier to professional recertification for newly arrived immigrants is a financial one. The tension between the resettlement costs and the fees required to have their credentials assessed and verified can be significant. Many IEHPs are forced into non-professional jobs to meet their immediate needs, which can have negative consequences for professional integration. Johnson and Baumal who investigated the professional integration of IEHPs found when IEHPs began working in “survival” jobs, it became far more difficult for them to re-enter their profession in Canada [21].

The second most frequently identified barrier is IEHPs’ lack of knowledge of how to navigate social and professional resources in their new country. IEHPs often lack information about the programs and resources available to initiate the credential verification and assessment process, how to secure housing that permits easy access to the available programs and resources, and initiate contact with the ethnic or cultural resources within their communities [22].

#### Policy solutions to ameliorate these challenges

The literature identifies both federal and provincial initiatives to support IEHPs during the early arrival period. For example, federal and provincial loans are available to help IEHPs offset the costs of having their credential verified and assessed [23]. Several provinces and territories have implemented microcredit programs to assist newcomers [24]. Provincially based IEHP specific settlement programs are available to provide information about the licensing process [25]. Jablonski found the use of a case management approach that provides support to IEHPs who are eligible, as well as those that are not yet eligible to practice, as effective for helping IEHPs navigate the professional integration process during the early phases of their resettlement period [26].

#### Role of professional associations

The review identified several professional associations that provide support and services to IEHPs [27–29]. There are many large professional associations for nurses and physicians and as such have the infrastructure to support the integration of IEHPs through various initiatives [30, 31]. The Canadian Society for Medical Laboratory Science provides information to assist internationally educated medical laboratory technicians (IEMLT) traverse the professional integration landscape [32]; however, there is less literature that describes the role of the professional associations for other allied health professions.

In summary, what is known from the literature about early arrival activities and programs is the importance of providing system navigation assistance and access to various forms of support. The use of a case management approach towards helping IEHPs navigate recertification landscape is promising practice. The literature does not fully explain the process or address the range of services offered by immigrant settlement organizations and for IEHPs in allied health professions. There is also very little information about whether the programs are successful in addressing the needs of IEHPs.

### Professional recertification

The most prolific theme in the literature is about the processes IEHPs engage in to meet the requirements for registration with a professional regulatory body in Canada (*n* = 226, 46 %). Forty-two percent (*n* = 96) of this literature was about IMGs and 21 % (*n* = 47, 21 %) was about IENs. The professional recertification literature is organized into two main subthemes: barriers and facilitators to professional recertification and programs and policies to facilitate IEHP professional recertification.

#### Barriers and facilitators of professional recertification

The first step in the process of professional recertification is having one’s credentials verified and assessed by a regulatory body. This process can be complicated by the fact that IEHPs often are not familiar with the system of professional recertification (particularly the division of responsibilities between federal and provincial bodies), may not have all necessary documentation or may not know where to send the documents for verification and assessment. The process of professional recertification can also be time consuming and costly. The amount of time it takes to qualify for examinations, study and pass can be considerable with the time away from the profession being a critical factor that will influence IEHPs’ ability to recertify.

Language fluency plays a significant role in IEHPs’ ability to traverse the professional recertification landscape. Without language fluency, IEHPs are unable to thoroughly understand the professional recertification process or benefit from the resources created to facilitate their recertification. Language fluency also plays a significant role in IEHPs’ ability to prepare and pass licensing examinations. Passing a language test is a standard requirement for licensure, although the test may vary and require different passing scores [33]. There is considerable debate in the literature about the best methods for testing IEHP language fluency and their ability to competently communicate when in the professional environment [34, 35]. Lafontant highlights the importance of providing opportunities for francophone IEHPs to become professionally recertified as they are in a good position to provide services to francophone minority communities [36].

Cultural competence, or familiarity with the culture and specifics of practice within a new country, has been found to be important for IEHPs to achieve success on professional examinations, complete professional training and communicate with patients [37]. While the issue of cultural competence may be more pressing for the diverse group of IEHPs whose cultural heritage is markedly different from the North American culture, Bourgeault and colleagues found that even those IEHs who arrive in Canada from countries with similar health care practices (e.g. United Kingdom, United States and South Africa) often find it difficult to immediately adjust to the Canadian way of professional practice [10]. Despite the literature’s recognition of IEHPs’ lack of cultural competence as a barrier to professional recertification, there is very little discussion in the literature about the potential benefits of the diverse cultural heritage and knowledge of additional languages IEHPs may bring with them or how these attributes can positively influence patient care and the Canadian health care system.

#### Programs and policy initiatives to facilitate professional recertification

The literature suggests that various stakeholders and policy makers are fully aware of the many challenges that exist in the process of credential verification and assessment process. It also identifies the work of various organizations that ensure IEHPs have timely access to accurate information about the process and support. For example, there are a number of “credentialing agencies” that provide the initial equivalency information for regulatory colleges or certification bodies across the country. Additionally, corresponding programs have been developed to provide the opportunity for IEHPs to demonstrate they have acquired the appropriate skills and knowledge through experience, as opposed to formal education [38–40]. There are two types of programs presented in the literature that are instrumental in facilitating IEHPs’ professional recertification: IEHP bridging programs and residency training programs for IMGs.

Bridging programs have been established to ameliorate the various barriers to professional recertification for IENs and internationally educated allied health professionals. The bridging program literature represents 4 % (*n* = 21) of the IEHP literature and is primarily focused on IENs (*n* = 8, 38 %). Some bridging programs assist with preparations for the national licensing or certification examinations whereas others truly bridge the competencies that IEHPs have with Canadian requirements. Other bridging programs prepare “practice-ready” IEHPs for immediate work integration. A small proportion of the bridging literature (*n* = 15, 3 %) focuses on the bridging programs and residency training available to immigrant physicians. Despite wide variation in the content and structure of bridging programs, they are often identified as promising practices for facilitating the integration of IEHPs.

Major barriers raised in the literature are the limited delivery of bridging programs outside of major urban centres, low enrolment capacity of many programs and isolated or temporary funding dedicated to these initiatives. Many professions have been able to increase both class size and accessibility through development of distance education modules [41, 42]. IEHPs also experience financial barriers to successful completion of a bridging program. Most programs are not covered by provincial post-secondary student loans schemes, and when funding is provided, it is usually limited to direct program costs (such as tuition, book and equipment costs) with no supplements for daily living expenses (such as rent, transportation, childcare, food). Upon completion of bridging programs, IEHPs have report a better knowledge of the culture of health care in Canada practice and improved communication skills. This draws attention to the need to better integrate bridging programs both within the professional infrastructure as well as inter-professionally.

We did not identify bridging programs specifically for IMGs, but rather the literature called attended to the difficulties IMG can have when attempting to access to residency. Although the number of residency positions allocated for (or occupied by) IMGs grew substantially in the past decade. IMGs are still considerably less likely to secure a residency position than Canadian Medical Graduates.

In summary, the professional recertification literature encompasses two general themes. First, there are a number of publications that describe how the professional recertification process is seen as confusing and opaque by IEHPs. Second, the literature points to a number of policies and programs that have been put in place to respond to these concerns; however, they mainly focus on IMGs and IENs. What is known about the bridging programs available to IEHPs in the allied health professions is limited. We also do not know if the available programs are fully addressing the identified concerns, as there is very limited information about their effectiveness.

### Alternate paths to professional integration

There is very little literature (*n* = 27, 5 %) that examines the alternative paths IEHPs take to achieve professional integration. The literature is mainly focused on alternate paths for IMGs (*n* = 10, 37 %) or is not profession specific (*n* = 8, 30 %). The literature centres on discussing alternate roles for IEHPs, such as IMGs becoming physician assistants [43] or IENs as unregulated live-in caregivers [44, 45]. No literature could be found that described programs or policy initiatives specifically designed to facilitate IEHP profession integration by way of alternate professions.

In summary, there is a gap in the literature about the alternate paths to professional integration. Given that we have many IEHPs that cannot professionally recertify due to the various barriers, more information about how IEHPs access their profession by ways of other professions is warranted.

### Workplace integration

Workplace integration is the process of IEHPs becoming members of a workgroup within an organization where they can use their professional knowledge and expertise. The thematic analysis revealed one quarter (*n* = 123) of the IEHP literature focuses on workforce integration. Three quarters of the workplace integration literature concentrates on IMGs (*n* = 53, 43 %) and IENs (*n* = 36, 29 %). It is organized into three main themes, practice profiles of IEHPs, barriers and facilitators to workplace integration and the role of employers.

#### Practice profile of IEHPs

IEHPs tend to reside and work in urban areas where they are closer to well established immigrant communities and can access integration and professional support services [46]. When IEHPs are recruited to fill the geographical gaps in the health workforce, long-term sustainability is negatively affected [47]. IEHP migration follows the same patterns as their Canadian counterparts, as they often move from less to more prosperous provinces and from rural to urban areas of the country [48].

#### Barriers and facilitators to workplace integration

IEHPs continue to experience challenges once they are employed. Language barriers, developing trustful relationships with patients and colleagues and adapting to new technology and professional culture are cited in the literature [21]. IEHPs also continue to experience discrimination in the workplace [49, 50]; however, it is unclear if this is because they are visible minorities or because they have foreign credentials. The literature suggests that formal leadership roles are not readily available to some IEHP groups, such as IENs. Communication challenges and marginalization of immigrants are reasons given in the literature [51].

The literature also contains information about the facilitators of IEHPs’ workplace integration. In addition to direct to work bridging programs, employer-sponsored orientation programs, collegial environments, mentoring and social support are cited as important factors that assist IEHPs with integrating into their workplaces [52, 53].

#### Role of employers

The literature reveals employers view IEHPs as both beneficial and unfavourable additions to their workplaces. For example, some authors highlight how IEHPs can assist with the provision of linguistically and culturally sensitive patient care [21], while others [18] report employers are uncertain if IEHPs have the knowledge and skills needed to be successful in the workplace. No literature could be located that compared IEHPs’ work performance to Canadian-educated health professionals.

In summary, the workplace integration literature mainly focuses on IEHP practice patterns, including location and sector of practice and does not provide information about what they do. As a result, the number of IEHPs who are unemployed or to what extent they are underemployed is unknown. The literature also reveals IEHPs experience another layer of barriers and discrimination on top of those identified in the professional recertification literature, but does not fully discuss the reasons why this occurs. Information about IEHPs after they have professionally integrated and their opportunities for career advancement is also lacking. The literature does include reports of many promising practices to facilitate IEHP workplace integration. It does however indicate employers can play a critical role in IEHP professional integration, yet very little information is available about employers’ hiring decisions as they related to IEHPs. Additionally, little information is available about the workplace integration of IEHPs in the allied health professions.

## Discussion

This review provides an overview of the scope and content of the Canadian academic and grey literature about the professional integration of IEHPs. The literature about IEHPs’ professional integration is abundant, mainly reports or journal articles and primarily pan-Canadian in scope. The majority of the literature is focused on the professional recertification of the two largest professions, nursing and medicine. Literature about IEHPs in the allied health professions is lacking. Empirical methods were used for nearly one half of the 407 publications, yet very little information is available about the effectiveness of the policies and programs available to facilitate IEHP integration.

### Amount, sources and distribution of literature

The high volume of publications reflects sustained policy relevance of the recruitment, recognition and professional integration for IEHPs. The literature also reflects the significant amount of work conducted to facilitate the professional integration of IENs and IMGs, professions with high numbers of IEHPs and that are in demand globally. The limited information about IEHPs in the allied health professions does not seem proportionate to the size of the profession or the numbers who migrate and as a result is troubling. There may be several reasons for this: First, the size of literature may reflect the lack of information about or absence of actual policy initiatives to facilitate integration of allied health professionals. Alternatively, it may simply indicate disinterest in this professional group in the literature particularly because allied health professionals are comprised of many different professional groups, some of which are regulated and some not. Plus, internationally educated allied health workers are not as vocal in public forums as other regulated professions in that they lack support that the larger professions such physicians and nurses enjoy through their affiliation with their professional associations. As such, relative less political advocacy may account for the reduced visibility in literature about the programs or policy initiatives available to facilitate the integration of allied health professions.

Close to half of the publications reported using empirical methods. This literature covers a wide range of topics and IEHP groups. The majority of this work is descriptive in nature or conducted with small samples sizes and may lack generalizability. Consideration could be given to exploring if the empirical literature about IEHPs is suitable for systematic reviews [4, 7, 54]. The empirical literature also tends to focus primarily on IEHPs who are already in the “system” and not about IEHPs who “opt-out” or are not pursuing professional recertification. This may be because identifying and approaching potential participants, especially IEHPs who have not professionally recertified and are not registered with a regulatory college, is especially challenging. Currently, data systems to track IEHPs’ adaption and integration are wanting. Developing databases to capture information about IEHPs would create information that could be used in decision-making and future research.

The available literature is geographically distributed across the provinces and territories of Canada. Still the literature is somewhat Ontario centric. The geographical distribution of the literature most likely reflects the funding sources used to develop the program or policy initiatives or to conduct the research. Ontario is one of the most populous provinces in Canada and as such has an active labour market and financial resources available to direct towards developing programs and policies to support the professional integration of IEHPs. For these reasons, many IEHPs are drawn to settle in Ontario.

The literature also tends to be profession specific and does not often consider the personal or professional attributes of IEHPs educated in the same country or region. IEHPs are not a homogenous group; they migrate to Canada from many different countries within different regions of the world. Since it is possible that not all IEHPs require the same types and levels of support conducting research about IEHPs from the same country or region may provide meaningful evidence to tailor current policy initiatives and programs to meet the needs of particular groups of IEHPs. This may prove to be a more “fruitful” method for addressing the learning and social needs of IEHPs thus facilitating their integration and retention [55].

### Alternate paths to IEHP professional integration

Possible explanations for the surprisingly limited knowledge about the alternate paths IEHPs pursue towards professional integration are that this potentially promising avenue has not been a significant focus for policy initiatives or program development. We identified one publication that discusses initiatives to retrain IEHPs for complementary regulated professions, e.g. IMGs to physician assistants [56]. No other information could be located about what alternate paths IEHPs take to professional integration. Another possible reason for the limited knowledge about the alternate paths to IEHPs’ professional integration could be that when IEHPs fail to recertify in their professions they choose to pursue roles outside of the regulatory health professions. The literature does point to IEHPs pursuing other health care roles that are not regulated, for example retraining internationally trained pharmacists for pharmacy assistants or pharmaceutical representatives [57]. It is possible that the related publications were not identified through our search strategy and thus not captured in our literature search. Understanding how to best utilize the human capital of the IEHPs who fail to professionally recertify is a promising area for research and policy development [58].

### Effectiveness of policy initiatives and programs

The possible reasons for the absence of studies to evaluate the effectiveness of the policy initiatives and programs to facilitate the professional integration of IEHPs could be the inherent time lag between their development and evaluation. In recent years, considerable financial investment, both public and private, has been devoted towards streamlining the recognition and integration of IEHPs. This has resulted in numerous promising policy initiatives and programs, which may not yet be evaluated. This signifies a need for studies to determine the effectiveness as well as the long-term outcomes of these initiatives.

### Strengths and limitations

This scoping review has several strengths including the use of methodologically sound and transparent process to identify and map the literature. First, a team of researchers who had expertise in the substantive area, knowledge syntheses and scoping review methodology conducted the review. Second, an exhaustive search strategy with several bibliographic databases, the Worldwide Web and stakeholders, was used to identify both the academic and grey literature. Third, the use of standardized screening and data extraction forms that were pilot tested prior to their use to extract and code the data, ensured consistency in the identification and coding of the literature. Fourth, stakeholders’ involvement and their contributions throughout the project made the findings applicable and highly relevant to stakeholders and knowledge-users.

There are some limitations. It is possible that despite the use of an exhaustive search strategy some literature, particularly the grey literature, could have been missed and thus not included in this review. Second, it is possible that some stakeholders were more active than others, and as a result, we may have missed the opportunity to include some evidence, particularly unpublished literature. And third, while the scoping review methodology allows for the inclusion of a wide range of evidence, it did not permit us to appraise the quality of the evidence [4, 7], which may have had some influence on the thematic analysis.

## Conclusions

Canada provides an excellent case for examining the literature about the professional integration of IEHPs. Other countries can take several things from our review. For example they may want to consider using a similar set of thematic criteria to categorize their literature on IEHP integration. The findings could be contrasted with the literatures of this review and IEHP integration literature in other destination countries. Stakeholders and policy-decision makers in other destination countries can reflect on the main conclusions in an assessment of their policies, programs and processes. Migrating health professionals as well as key policy-decision makers in key source countries can similarly reflect on the key findings for how IEHPs fare in Canada, for more informed personal and policy decisions.

## Abbreviations

IEHPs, internationally educated health professional-no specific profession; IEMLTs, internationally educated medical laboratory technologist; IEMRTs, internationally educated medical radiation technologists; IEMs, internationally educated midwives; IENs, internationally educated nurses; IEOTs, internationally educated occupational therapists; IEPTs, internationally educated physical therapists; IERTs, internationally educated respiratory therapists; IMGs, international medical graduates; ITDs, internationally trained dentists; ITPs, internationally trained pharmacists

## Additional files

Additional file 1: Sources retained for data extraction and charting. (PDF 352 kb) Additional file 2: Thematic coverage per source. (PDF 596 kb)
