# Supplementary material for: Scoping review about the professional integration of internationally educated health professionals
Source: Hum Resour Health. 2016 Jun 17;14:38. doi: 10.1186/s12960-016-0135-6 (PMC4912807; doi:10.1186/s12960-016-0135-6)
Supplement: Additional file 1: — Sources retained for data extraction and charting. (PDF 352 kb) [file 12960_2016_135_MOESM1_ESM.pdf]

Additonal Table 1
Sources Retained for Data Extraction and Charting
*** Cited in Review**

1. Advisory Committee on Health Delivery and Human Resources [ACHDHR]: **Self sufficiency - More Than Numbers: A Canadian Perspective.** In *International Medical Workforce Collaborative 2010*; 2010.
-

2. *Agbassi I, Don-Wauchope, A.C.: **Challenges facing internationally educated medical laboratory technologists in Ontario-a pilot study.** *Canadian Journal of Medical Laboratory Technologist* 2010, **72:**88-89.

3. * Alberta International Medical Graduates: **Alberta International Medical Graduates Association [AIMGA]. Website.** [Retrieved from: http://aimga.ca]

4. Alberta Network of Immigrant Women [ANIW]: **Access to Licensure for Foreign Qualified Women.** Edmonton, Alberta; 2002.

5. *Alberta Network of Immigrant Women [ANIW]: **Access to licensure for internationally educated nurses: Follow up study.** Edmonton, Alberta; 2005.

6. Andrew RF: **How do IMGs compare with Canadian medical school graduates in a family practice residency program?** *Canadian Faimly Physician* 2010, **56:**e318-322.

7. Association of Canadian Community Colleges: **Meeting Expectations: A Blueprint for Sustaining the Allied Helath Professions.** Ottawa, Ontario: Association of Canadian Community Colleges; 2012.

8. Association of Faculties of Medicine of Canada [AFMC]: **The National IMG Database Report.** Ottawa, Ontario; 2011.

Additonal Table 1
Sources Retained for Data Extraction and Charting
*** Cited in Review**

9. Association of International Physicians & Surgeons of Ontario [AIPSO]: **Barriers to Licensing in Ontario for Internationanl Physicians.** Toronto, Ontario; 2000

10. Association of International Physicians & Surgeons of Ontario [AIPSO]: **Integrating Canada's Internationally-Trained Physicians: Towards a Coherent, Equitable and Effective National System.** Toronto, Ontario, Canada: The Commission on the Future of Healthcare in Canada; 2002.

11. *Association of International Physicians and Surgeons of Ontario: **Internationally Trained Medical Doctors in Canada.** vol. 2013; 2012.

12. Atack L, Cruz, E. V., Maher, J., Murphy, S.: **Internationally educated nurses' experiences with an integrated bridge program.** *Journal of continuing education in nursing* 2012, **43:**370.

13. Atlin J: **Integrating Canada's internationally-trained physicians: towards a coherent, equitable and effective national system.** *International Settlement Canada Research Resource Division for Refugees* 2002, **16:**6-10.

14. Audas R, Ross., A. Vardy, D.: **The Role of International Medical Graduates in the Provision of Physician Services in Atlantic Canada.** St. John's, NL Harris Centre of Regional Policy and Development & Faculty of Medicine, Memorial University of Newfoundland Queens College 2004.

15. Audas R, Ross., A. Vardy, D.: **The use of provisionally licensed internation medical graduates in Canada.** *CMAJ: Canadian Medical Association Journal* 2005, **173:**1315-1316

Additonal Table 1
Sources Retained for Data Extraction and Charting
*** Cited in Review**

16. Audas R, Ryan, A., Vardy, D.: **Where did the doctors go? A study of retention and migration of provisionally licensed international medical graduates practising in Newfoundland and Labrador between 1995 and 2006.** *Canadian Journal of Rural Medicine* 2009, **14:**21-24.

17. *Austin Z: **Continuous Professional Development and Foreign-Trained Health Professionals. Results of an Educational Needs Assessment of International Professional Graudates in Ontario (Canada).** *Journal of Social and Administrative Pharmacy* 2003, **20:**232-241.

18. Austin Z: **Mentorship and mitigation of culture shock: foreign-trained pharmacists in Canada.** *Mentorship and Tutoring* 2005, **13:**133-149.

19. Austin Z, Rocchi Dean, M: **Bridging Education for Foreign-Trained Professionals: the International Pharmacy Graduate (IPG) Program in Canada.** *Teaching in Higher Education* 2006, **11:**19-32.

20. Austin Z, Croteau, D.: **Intersectoral collaboration to enable bridging education for pharmacists: The International Pharmacy Graduate Program in Ontario, Canada.** *Pharmacy Education* 2007, **7:**61-68.

21. Austin Z, Martin, J.C., Gregory, P.A.M.: **Estimation of financial returns on investment in bridging education in pharmacy.** *Pharmacy Education* 2007, **7:**133-139.

22. Austin Z: **Geographical migration, psychological adjustment, and re-formation of professional identify: the double-culture shock experience of international pharmacy graduates in Ontario.** *Globalisation, Societies and Education* 2007, **5:**239-255.

23. *Austin Z: **Bridging to Success: A Learning Day About Bridging Programs in Regulated Professions.** Ontario, Canada: Ontario Regulators for Access Consortium; 2008. http://www.regulatorsforaccess.ca/docs/Bridging_Success.pdf

Additonal Table 1
Sources Retained for Data Extraction and Charting
*** Cited in Review**

24. Austin Z, Ensom, M.H.H.: **Education of pharmacists in Canada.** *American Journal of Pharmaceutical Education International Pharmacy Education Supplement* 2008, **72** 1-11 (Article 128).

25. *Austin Z, Galli M, Diamantouros A: **Development of a prior learning assessment for pharmacists seeking licensure in Canada.** *Pharmacy Education* 2003, **3:**87-96.

26. Austin Z, Gregory PAM, Galli M: **"I just don't know what I'm supposed to know". Evaluating self-assessment skills of international pharmacy graduates in Canada.** *Research in Social and Administrative Pharmacy* 2008, **4:**115-124.

27. Baerlocher MO: **The importance of foreign-trained physicians to Canada.** *Clinical and Investigative Medicine* 2006, **29:**151-153.

28. Baig LA, Violato, C., Crutcher, R. A.: **Assessing clinical communication skills in physicians: are the skills context specific or generalizable.** *BMC Medical Education* 2009, **9**. http://link.springer.com/article/10.1186%2F1472-6920-9-22#/page-1

29. Baldacchino G, Hood, M.: **Challenges Faced by Internationally Educated Health Professionals on Prince Edward Island: Stories and Voices: A Research Report for IEHP Atlantic Connection.** IEHP Atlantic Connection; 2008. http://www.islandstudies.ca/sites/vre2.upei.ca.islandstudies.ca/files/4/IEHP_Study-Final-Feb2008.pdf

30. Baldacchino G, Hood, M.: **Challenges faced by Internationally Educated Health Professionals on Prince Edward Island. A Research Report for IEHP Atlantic Connection.** IEHP Atlantic Connection; 2008. http://www.atlanticcanadahealthcare.com/images/pdf/Stories__Voices_PE_08.pdf

31. Baldacchino G, Saunders, P.: **Internationally Educated Health Professionals in Nova Scotia and Prince Edward Island. Why They Come, Why They Stay and the Challenges They Face: A Follow-Up Study.**; 2010. http://www.atlanticcanadahealthcare.com/images/pdf/Stories__Vocies_NL_08.pdf

Additonal Table 1
Sources Retained for Data Extraction and Charting
*** Cited in Review**

32. Banerjee L: **Supervision of internationally educated nurses (IENs) graduate nurses.** *SRNA Newsbulletin* 2010, **12:**19-19.
33. Banner S, Bowmer, M.I., Rattanasithy, S.: **Unrecognized Health Human Resource Impact of Canadians Studying Medicine Abroad (CSAs). Poster Presentation.** In *13th International Health Workforce Collaborative*. October 24–26, Brisbane, Australia: Royal College of Physicians and Surgeons of Canada; 2011.
34. Baptiste SE, Blais, P., Brenchley, C.L., Sauve, D.E., McMahon, P.A.: **Supporting (re) entry to professional practice: The SEPP project.** *Canadian Journal of Occupational Therapy* 2010, **77:**144-150.
35. Bard R: **[Towards a balance between security and mobility of nurses in Canada].** *Soins; la revue de reference infirmiere* 2009**:**45-47.
36. Baringhausen T, Bloom, D.E.: **Changing Research Perspectives on the Global Health Workforce** Cambridge, MA: National Bureau of Economic Research; 2009. http://www.nber.org/papers/w15168
37. Barry J, Sweatman, L., Little, L., Davies, J.: **International nurse applicants.** *Canadian Nurse* 2003, **99:**34-35.
38. Bassendowski S, Petrucka, P.: **A Consideration of Transition for Internationally Educated Nurses in Saskatchewan IEN Research Project.** 2010.
39. Bates J, Andrew, R.: **Untangling the Roots of Some IMGs' Poor Academic Performance.** *Academic Medicine: Journal of the Association of American Medical Colleges* 2001, **76:**43-46.

Additonal Table 1
Sources Retained for Data Extraction and Charting
*** Cited in Review**

40. Baumann A, Blythe, J., Kototylo, C.,: **Immigration and Emigration Trends: A Canadian Perspective.** In *Building the Future: An Integrated Strategy for Nursing Human Resources in Canada*. Ottawa, Ontario, Canada: Nursing Health Services Research Unit; 2004. http://tools.hhr-rhs.ca/index.php?option=com_mtree&task=att_download&link_id=5292&cf_id=68&lang=en

41. Baumann A, Blythe, J., Kolotylo, C., Underwood, J.: **Mobility of Nurses in Canada.** In *Building a Future: An Integrated Strategy for Nursing Human Resources in Canada* (Downey M ed. Ottawa, Ontario: Canadian Nurses Association; 2004.

42. *Baumann A, Blythe, J., Kolotylo, C.: **Building the Future: An Integrated Strategy for Nursing Human Resources in Canada: A Discussion Paper.** Ottawa, ON, Canada; 2006.

43. Baumann A, Blythe, J.: **Integrating Internationally Educated Health Professionals into the Ontario Workforce.** (Association OH ed. pp. 66: Nursing Health Services Research Unit; 2009:66. https://www.oha.com/Services/HealthHumanResources/EmployingIEHP/Documents/Integrating%20IEHP%20into%20the%20Ontario%20Workforce%20FULL%20REPORT%20Dec%202009.pdf

44. Baumann A, Blythe, J.: **Community Collaboration for IEN/ESL Employment: Bridging the Gap: A Descriptive Evaluation.** 2012. https://books1.scholarsportal.info/viewdoc.html?id=693086

45. Baumann A, Blythe, J.: **Recruiting and Retaining Internationally Educated Nurses.** In *Evidence Note* Hamilton, Ontario: Nursing Health Sciences Research Unit; 2012. . http://nhsru.com/publications/evidence-note-on-recruiting-and-retaining-internationally-educated-nurses/

46. Baumann A, Blythe J, Hunsberger M: **How Government Invests in Research to Advance Policy: Evolution of Evidence** Hamilton, Ontario, Canada: Nursing Health Services Research Unit, McMaster University; 2010. . http://tools.hhr-rhs.ca/index.php?option=com_mtree&task=att_download&link_id=7235&cf_id=68&lang=en

Additonal Table 1
Sources Retained for Data Extraction and Charting
*** Cited in Review**

47. *Baumann A, Blythe J, Rheaume A, McIntosh K: **Internationally Educated Nurses in Ontario: Maximizing the brain gain** In *Human Health Resources Series*, vol. 2, 2 edition. pp. 46; 2006:46. http://nhsru.com/publications/internationally-educated-nurses-in-ontario-maximizing-the-brain-gain-2/

48. Baumann A, Blythe J, Ross D: **Internationally educated health professionals: workforce integration and retention.** *Healthcare Papers* 2010, **10**.

49. BCMA Council on Health Economics and Policy (CHEP): **Doctors Today and Tomorrow. Planning British Columbia's Physician Workforce.** In *A Policy Paper by BC's Physicians*. British Columbia, Canada; 2011. https://www.doctorsofbc.ca/sites/default/files/physicianworkforce_paper_web.pdf

50. Beaton M, Walsh, J.: **Overseas recruitment: Experiences of nurses immigrating to Newfoundland and Labrador 1949-2004.** *Nursing Inquiry* 2010, **17**.

51. Beck R, Haworth-Brockman, M., Martin, W., Kilthei, J. Rach, D., Erickson, J., Klick, K., Kilpatrick, R., Moise, M.: **The National Midwifery Assessment Strategy: Building Bridges.** *Canadian Journal of Midwifery Research and Practice* 2008, **7**:31-35.

52. Belkhodja C. FE, Gaboury I, Guignard Noel J., Bahi B., Nkolo C., Tawil, N.: **L'integration des diplômes internationaux en santé francophones dans les communautés francophones en situation minoritaire. Report final de recherché.** Ottawa, Ontario: Consortium national de formation en sante (CNFS); 2009. http://cnfs.net/wp-content/uploads/2015/06/rapport-final-10septv2.pdf

53. Beran T, . Violato, E., Faremo, S. Violato, C., Watt, D., Lake, D. : **Ego identity development in physicians: a cross-cultural comparison using a mixed method approach.** *BMC Research Notes* 2012, **5**:249.

Additonal Table 1
Sources Retained for Data Extraction and Charting
*** Cited in Review**

54. *Bhimji A: **International medical graduates: the multi-centres experience.** *Healthcare Papers* 2010, **10:**46-49.

55. Blais P: **Access Issues Regarding Internationally Educated Health Professionals and the Respiratory Therapy Profession in Canada.** Montreal, Quebec, Canada: National Alliance of Respiratory Therapy Regulatory Bodies (NARTRB); 2008. http://www.nartrb.ca/eng/documents/NAReportEnglishFinalApril4.pdf

56. Blais P, Darling, P.: **An Analysis of the Performance of Internationally Educated Medical Radiation Technologist (IEMRTs) on the CAMRT Radiological Technology Certification Examination.** (Canadian Association of Medical Radiation Technologists (CAMRT) ed. Ottawa, Ontario: Canadian Association of Medical Radiation Technologist (CAMRT); 2009.

57. Blooberg NB, Schönwetter, D.J., Swain, V.L.: **Advanced placement, qualifying, and degree completion programs for internationally trained dentists in Canada and the United States: an overview.** *Journal of Dental Education* 2009, **73:**399-415.

58. *Blythe J, Baumann A: **Supply of Internationally Educated Nurses in Ontario: Recent Developments and Future Scenarios** In *Health Human Resources Series*, vol. Health Human Resources. Hamilton Ontario: McMaster University; 2008.

59. Blythe J, Baumann A: **Internationally educated nurses: profiling workforce diversity.** *International Nursing Review* 2009, **56:**191-197.

60. *Blythe J, Baumann A, Rheaume A, McIntosh K: **Nurse migration to Canada: pathways and pitfalls of workforce integration.** *Journal of Transcultural Nursing* 2009, **20:**202-210.

Additonal Table 1
Sources Retained for Data Extraction and Charting
*** Cited in Review**

61. *Bobrosky W: **Towards Creating a Fair and Equitable Fast Track Assessment Process for Alberta International Medical Graduates** Calgary, Alberta, Canada; 2010.

62. Boschma G, Santiago, M.: **Health Worker mMigration in Canada: Histories, Geographies and Ethics.** In *Working Paper Series* (Sheldon L ed. British Columbia: Metropolis British Columbia Centre of Excellence for Research on Immigration and Diversity; 2012. http://mbc.metropolis.net/assets/uploads/files/wp/2012/WP12-02.pdf

63. Bourgeault IL: **On the Move: The Migration of Health Care Providers in Canada** [Retrieved from: http://www.ic.gc.ca/epic/site/eas-aes.nsf/en/ra01976e.html]

64. Bourgeault IL: **Health care brain waste needs a long-term policy solution.** In *Embassy- Canada's Foreign Policy Newspaper*; 2007. http://www.embassynews.ca/news/2007/03/28/health-care-brain-waste-needs-a-long-term-policy-solution/34858

65. Bourgeault IL: **If the answer is "more nurses," what is the question?** *Canadian Nurse* 2012, **108:**44.

66. Bourgeault IL, Atanackovic J, LeBrun J, Parpia R, Rashid A, Winkup J: **The Role of Immigrant Care Workers in an Aging Society. The Canadian Context & Experience.** 2009.

67. Bourgeault IL, Baumann A: **Ethical recruitment and integration of internationally educated health professionals in Canada** In *13th International Health Workforce Collaborative 2011*: Royal College of Physicians and Surgeons of Canada; 2011.

Additonal Table 1
Sources Retained for Data Extraction and Charting
*** Cited in Review**

68. Bourgeault IL, Neiterman E, Le Brun J: **Midwives on the move: Comparing the requirements for practice and integratin contexts for internationally educated midwives in Canada with U.S., U.K. and Australia.** *Midwifery* 2011, **27:**368-375.

69. *Bourgeault IL, Neiterman E, LeBrun J, Viers, K., Winkup J: **Brain Gain, Drain and Waste: The Experiences of Internationally Educated Health Professionals in Canada.** Ottawa, Ontario: University of Ottawa; 2010. http://www.threesource.ca/documents/February2011/brain_drain.pdf

70. Bourgeault IL, Parpia R, Neiterman E, Le Blanc Y, Jablonski J: **Immigration and HHR Policy Contexts in Canada, the U.S., the U.K. & Australia.** 2011. http://rcpsc.medical.org/publicpolicy/documents/2011/IHWC_Canada_Theme.pdf

71. Bowmer I: **Canadian Initiatives: Assessment and integration of International Medical Graduates and Other Internationally Educated Health Professionals.** In *International Medical Workforce Collaborative 2005*. Melbourne Australia: Royal Colleges of Physicians and Surgeons of Canada; 2009. . http://rcpsc.medical.org/publicpolicy/ihwc.php

72. Boyd M, Schellenberg, G.: **Re-accreditation and the occupations of immigrant doctors and engineers.** In *Canadian Social Trends*. pp. 2010. Ottawa, Ontario: Statistics Canada; 2008:2010. http://www.statcan.gc.ca/pub/11-008-x/2007004/10312-eng.htm.

73. British Columbia Health Professions Review Board Stakeholder Consultation Committee: **Best Practices Pilot Study on Health Professions Registration.** British Columbia; 2010. http://www.hprb.gov.bc.ca/publications/Best_Practices_Pilot_Study.pdf

74. *Broten L: **Report on Removing Barriers for International Medical Doctors.** Toronto, Ontario, Canada: Ontario Ministry of Health and Long-Term Care; 2008. http://www.health.gov.on.ca/en/common/ministry/publications/reports/removing_barriers/removing_barriers.aspx

Additonal Table 1
Sources Retained for Data Extraction and Charting
*** Cited in Review**

75. Brown TA, Raborn, W.: **Is there an adequate supply of new dentists in Canada?** *Journal of the Canadian Dental Association* 2001, **67:**373-374.

76. Brownell AK: **Clinical neurology training of foreign nationals in Canada--the current situation.** *The Canadian Journal of Neurological Sciences* 2011, **38:**539-540.

77. Bruce D, Zwicker, G.: **Internationally educated health professionals in New Brunswick: Why they come, why they stay and the challenges they face.** Sackville, NB: Mount Allison University; 2008. http://www.atlanticcanadahealthcare.com/images/pdf/Stories__Vocies_NL_08.pdf

78. Brynaert and Associates: **Étude ciblée de la situation des diplômés internationaux en santé à Toronto et dans le Sud-ouest de l'Ontario.** Ottawa, ON, CAN; 2010. . http://cnfs.net/wp-content/uploads/2015/06/--tude-cibl--e-PFSFE-Toronro-et-sud-est-ON.pdf

79. Buchan J: **Nurses moving across borders: 'Brain drain' or freedom of movement? .** *International Nursing Review* 2001, **48:**65-67.

80. Buhr KJ: **Three Essays on the Labour Market For Registered Nurses in Canada.** Carelton University, Economics; 2006.

81. Buske L: **Trainees sponsored by foreign governments fill growing share of residency slots.** *Canadian Medical Association Journal* 2000, **163:**1185.

Additonal Table 1
Sources Retained for Data Extraction and Charting
*** Cited in Review**

82. Buske L, Slade, S.: **Tracking practice entry cohorts of Canadian post-MD education programs | Suivi des cohortes de nouveaux praticiens dans le cadre des programmes canadiens de formation post doctorale.** Ottawa, Onatrio: AFMC; 2009. . https://www.cma.ca/Assets/assets-library/document/en/advocacy/43-Bulletin_tracking_postgraduate_practice_entry_cohorts_e.pdf

83. Buske L: **Number of "Canadian IMGs" seeking residency training soars.** Ottawa, Ontario: Canadian Medical Association; 2010. https://www.afmc.ca/pdf/datapoint/DATAPOINT-may-eng.pdf

84. Cadieux G, Tamblyn, R., Dauphinee, D., Libman, M.: **Predictors of inappropriate antibiotic prescribing among primary care physicians.** *CMAJ: Canadian Medical Association Journal* 2007, **177**.

85. Cameron PJ, Este, D.C., Worthington, C.A.: **Physician retention in rural Alberta: key community factors.** *Canadian Journal of Public Health* 2010, **101:**79-82.

86. Canadian Alliance of Physiotherapy Regulators and Canadian Physiotherapy Association: **Physiotherapy Health Human Resources. Background Paper.** Ottawa, Ontario: Health Canada Health Human Resource Strategies Division, 2002. http://tools.hhr-rhs.ca/index.php?option=com_mtree&task=att_download&link_id=5047&cf_id=68&lang=en

87. Canadian Association of Medical Radiation Technologists: **National Guidelines for the Assessment of Credentials of Internationally Educated Medical Radiation Technologists.** 2012. www.camrt.ca

88. Canadian Association of Medical Radiation Technologists [CAMRT]: **A Situational Analysis and Recommendations for Internationally Educated Medical Radiation Technologists I.** Ottawa, Ontario. 2006.

89. Canadian Association of Occupational Therapist: **The Development of an Access and Registration Framework for Internationally Educated Occupational Therapists: Summary Report 2007.** 2007. https://www.caot.ca/pdfs/ieot%20summary.pdf

Additonal Table 1
Sources Retained for Data Extraction and Charting
*** Cited in Review**

90. Canadian Assoication of Occupational Therapists: International Educated Occupational Therapists, 2012. http://www.caot.ca/default.asp?pageid=2195.

91. Canadian Association of Physiotherapists Regulators: **Canadian Alliance of Physiotherapy Regulators (The Alliance) ,** http://www.alliancept.org]

92. Canadian Association of Schools of Nursing: **Final Report on the Pan-Canadian Framework of Guiding Principles and Essential Components for IEN Bridging Programs.** Ottawa, Ontario; 2012. http://www.casn.ca/en/Competencies_and_Indicators_168/items/2.html

93. Canadian Collaborative Centre for Physician Resources and Canadian Medical Association: **International Medical Graduates in Canada.** Ottawa, Ontario; 2008.

94. Canadian Federation of Nurses Unions (CFNU): **A Position Statement on Internationally Educated Nurses.** 2009. https://nursesunions.ca/sites/default/files/Internationally_Educated_Nurses_Position_Statement.pdf

95. Canadian Institute for Health Information (CIHI): **Regulated Nurses: Canadian Trends, 2007 to 2011.** Ottawa, Ontario; 2011. https://secure.cihi.ca/free_products/Regulated_Nurses_EN.pdf

96. Canadian Institute for Health Information (CIHI): **Regulated Nurses: Canadian Trends, 2006 to 2010.** Ottawa, ON, CAN: Canadian Institute for Health Information; 2012. https://secure.cihi.ca/free_products/RegulatedNurses2012Summary_EN.pdf

97. Canadian Institute for Health Information (CIHI): **Summary report: Distribution and Internal Migration of Canada's Health Care Workforce** Ottawa, Ontario: Canadian Institutes of Health Information; 2007. https://secure.cihi.ca/free_products/2007_RN_EN_web.pdf

Additonal Table 1
Sources Retained for Data Extraction and Charting
*** Cited in Review**

98. Canadian Institute for Health Information (CIHI): **Workforce Trends of Registered Nurses in Canada, 2005,.** vol. 2007. Ottawa, Ontario; 2007.

99. Canadian Institute for Health Information (CIHI): **Internationally educated physicians and nurses in Canada** Ottawa, Ontario: Canadian Institute of Health Information; 2007. http://www.cihi.ca/CIHI-ext-portal/internet/EN/Home/home/cihi000001.

100. Canadian Institute for Health Information (CIHI): **Canada's Health Care Providers, 2007.** Ottawa, Ontario: Canadian Institutes for Health Information; 2007. https://secure.cihi.ca/free_products/HCProviders_07_EN_final.pdf

101. Canadian Institute for Health Information (CIHI): **Health Care in Canada, 2007.** Ottawa, Ontario, Canada: Canadian Institutes for Health Information; 2007. https://secure.cihi.ca/free_products/hcic2007_e.pdf

102. Canadian Institute for Health Information (CIHI): **Canada's Health Care Providers, 1997 to 2006. A Reference Guide.** Ottawa, Ontario: Canadian Institute of Health Information; 2008. https://secure.cihi.ca/free_products/HealthCareProv_RefGuideEN_Final_Sep18.pdf

103. Canadian Institute for Health Information (CIHI): **Regulated Nurses: Trends, 2003 to 2007; Registered Nurses, Licensed Practical Nurses, Registered Psychiatric Nurses.** Ottawa, ON, Canada: Canadian Institute for Health Information; 2008. https://secure.cihi.ca/free_products/Regulated_Nurses_EN.pdf

104. Canadian Institute for Health Information (CIHI): **Regulated Nurses: Canadian Trends, 2004 to 2008.** Ottawa, ON, CAN: Canadian Institute for Health Information; 2009.

105. Canadian Institute for Health Information (CIHI): **International Medical Graduates in Canada: 1972 to 2007.** Ottawa, Ontario; 2009. https://secure.cihi.ca/free_products/img_1972-2007_aib_e.pdf

Additonal Table 1
Sources Retained for Data Extraction and Charting
*** Cited in Review**

106. Canadian Institute for Health Information (CIHI): **Regulated Nurses: Canadian Trends, 2005 to 2009.** Ottawa, ON, CAN: Canadian Institute for Health Information; 2010. https://secure.cihi.ca/free_products/RegulatedNursesCanadianTrends2006-2010_EN.pdf

107. Canadian Institute for Health Information (CIHI): **Occupational Therapists in Canada.** Ottawa, Ontario, Canada; 2012. https://www.cihi.ca/en/ot2011_highlights_profiles_en.pdf

108. Canadian Institutes for Health Information: **Supply, Distribution and Migration of Canadian Physicians. Methodological Notes.** Ottawa, Ontario, Canada; 2012. https://secure.cihi.ca/free_products/SMDB_2009_EN.pdf

109. Canadian Labour and Business Centre: **Physician Workforce in Canada: Literature Review and Gap Analysis** In *Task Force Two: A Physician Human Resource Strategy for Canada*. Ottawa, Ontario; 2003. http://www.saglikpolitikalari.org/images/dosyalar/physicianworkforcecanada.pdf

110. Canadian Medical Association (CMA): **Who Has Seen The Winds of Change? Toward a Sustainable Canadian Physician Workforce.** Ottawa, Ontario: CMA Ad Hoc Policy Working Group on the Physician Workforce; 2004.

111. Canadian Medical Laboratory Technologist Society (CMLTS): **Language Proficiency Testing for Internationally Educated Medical Laboratory Technologist: Validating Cut Scores and a New Testing Tool. CSMLS Project Report.** Hamilton, Ontario: Canadian Society for Medical Laboratory Science; 2009. http://csmls.org/csmls/media/documents/publications/reports/CSMLS_Project_Report_to_Ministry_-_Final.pdf

112. Canadian Nurses Association: **Sustaining the Workforce by Embracing Diversity.** Ottawa, ON, Canada; 2009. . https://cna-aiic.ca/~/media/cna/page-content/pdf-en/hhr_policy_brief5_2009_e.pdf?la=en

Additonal Table 1
Sources Retained for Data Extraction and Charting
*** Cited in Review**

113. Canadian Nurses Association (CNA) and Canadian Medical Association (CMA): **Towards a Pan-Canadian Planning Framework for Health Human Resources: A Green Paper.** Ottawa, Ontario, Canada: Canadian Nurses Association; 2005. . https://www.cna-aiic.ca/~/media/cna/page-content/pdf-fr/cma_cna_green_paper_e.pdf?la=en

114. Canadian Nurses Association [CNA]: **Position Statement. International Trade and Labour Mobility.** Canadian Nurses Association; 2009. https://www.cna-aiic.ca/~/media/cna/page-content/pdf-en/ps103_international_trade_e.pdf?la=en

115. Canadian Post- M.D. Education Registry: **National IMG Database: Tracking the Acquisition of Canada Credentials and Access to Practice. Preliminary Report.** 2006.

116. Canadian Society for Medical Laboratory Science [CSMLS]: **Needs Assessment: Peer Support Network for Internationally Educated Medical Laboratory Technologists** 2011. http://csmls.org/csmls/media/documents/publications/reports/PeerSupportFinalReportNov302011.pdf

117. Canadian Society of Medical Laboratory Sciences: **Backgrounder: The CSMLS Prior Learning Assessment Process.** *Canadian Journal of Medical Laboratory Sciences* 2009, **71:**190-191.

118. Carter T, Morrish, M., Amoyaw, B.: **Attracting immigrants to smaller urban and rural communities: Lessons learned from the Manitoba Provincial Nominee Program.** *International Migration and Integration* 2008, **9:**161-183.

119. Carter T, Polevycho, C., Osborne, J., Adeler, M., Friesen, A.: **An Evaluation of the Manitoba Provincial Nominee Program: Prepared for Manitoba Labour and Immigration Division.** Winnipeg, Manitoba: The University Of Winnipeg; 2009.

Additonal Table 1
Sources Retained for Data Extraction and Charting
*** Cited in Review**

120. Cartmel V: **Assessing an applicant's competencies to practice.** *Nursing BC / Registered Nurses Association of British Columbia* 2009, **41**.

121. Cayabyab A: **Working toward success: Studying for the CRNE.** *SRNA NewsBulleting* 2010, **Winter:**12.

122. Centre for Internationally Educated Nurses (CARE): **A Decade of Service, A Decade of Success: Annual Report 2011-2012.** Toronto, Ontario: CARE. Centre for Internationally Educated Nurses 2012. http://care4nurses.org/wp-content/uploads/CARE_Annual_Report_2011-2012.pdf

123. Centre for the Evaluation of Health Professionals Educated Abroad [CEHPEA]: **Centre for the Evaluation of Health Professionals Educated Abroad (CEHPEA)-Annual Report 2011/12.** Toronto, Ontario: Centre for the Evaluation of Health Professionals Educated Abroad (CEHPEA); 2012. http://www.touchstoneinstitute.ca/Touchstone/media/Annual-Reports/AnnualReport_2011_12.pdf

124. Chan BTB: **From Perceived Surplus to Perceived Shortage: What Happened to Canada's Physician Workforce in the 1990s? .** Ottawa, Ontario, Canada: Canadian Institutes of Health Information; 2002. https://secure.cihi.ca/free_products/chanjun02.pdf

125. Chew D, Amirthalingam, V., Firoz, T., Goyal, A., Singh, J.: **International Medical Graduates: The BC Doctor Shortage Solution.** *UBCMJ University of British Columba Medical Journal* 2010, **1:**41-42.

126. Childs R, Herbert, M.: **Assessing IMG Performance at Ontario Medical Schools 2002-2006.** Ontario Institute for Studies in Education of the University of Toronto for the Council of Faculties of Medicine of the Council of Ontario Universities; 2007.

Additonal Table 1
Sources Retained for Data Extraction and Charting
*** Cited in Review**

127. Cho S, Masselink, L.E., Jones, C.B., Mark, B.A.: **Internationally Educated Nurse Hiring: Geographic Distribution, Community, and Hospital Characteristics.** *Nursing Economic$* 2011, **29:**308-316.

128. Choi LL: **Literature review: issues surrounding education of English-as-a-Second Language (ESL) nursing students.** *Journal of Transcultural Nursing* 2005, **16:**263-268.

129. Cholakis E: **Internationally trained dentists in Canada: an alternative view.** *Journal of the Canadian Dental Association* 2005, **71:**386-387.

130. Citizenship and Immigration Canada: **Strengthening Canada's Economy. Government of Canada Progress Report 2011 on Foreign Credential Recognition.** Government of Canada; 2011. http://www.cic.gc.ca/english/resources/publications/fcro/progress-report2011.asp

131. Citizenship and Immigration Canada [CIC]: **Facts and Figures 2011-Immigration Overview: Permanent and Temporary Residents** [Downloaded April, 24, 2013 from: http://www.cic.gc.ca/english/resources/statistics/facts2011/index.asp]

132. Coffey S: **Educating international nurses: curricular innovation through a bachelor of science in nursing bridging program.** *Nurse Educator* 2006, **31**.

133. College of Family Physicians of Canada: **Family Medicine in Canada. Vision for the Future.** Mississauga, Ontario, Canada; 2004.

134. College of Physicians and Surgeons of Ontario: **Tackling the Doctor Shortage. A Discussion Paper.** Ottawa, Ontario; 2004.

Additonal Table 1
Sources Retained for Data Extraction and Charting
*** Cited in Review**

135. College of Physicians and Surgeons of Ontario [CPSO]: **2006 Physician Resources in Ontario: Small Triumphs, Big Challenges.** Toronto, Ontario; 2007.

136. College of Physicians and Surgeons of Ontario [CPSO]: **Alternative Pathways to Registration.** Ontario, Canada: The College of Physicians and Surgeons of Ontario; 2008.

137. College of Registered Nurses of Nova Scotia: **Clearing the path for internationally educated nurses.** *Nursing in Focus* 2010, **Spring 2010:**9-11.

138. College of Registered Nurses of Nova Scotia: **Challenges faced by Internationally educated nurses call for innovative new strategies.** *Nursing Focus* 2010, **Fall 2010:**13-15.

139. Collins E: **Career Mobility Among Immigrant Registered Nurses in Canada: Experiences of Caribbean Women.** Ontario Institute for Studies in Education, University of Toronto, Department of Sociology and Equity Studies in Education; 2004.

140. Comeau M: **Physician Movement of Graduates from Canadian Medical Schools and International Medical Graduates.** Ottawa, Ontario; 2009.

141. Consortium National de Formation en Sante: **Linguistic Equity in Assessing the Credentials and Competencies of Internationally Educated Francophone Health Professionals.** Ottawa, Ontario; 2008.

142. Consortium National de Formation en Sante (CNFS): **The Recognition of Internationally Educated Francophone Health Professionals: Discussion Paper.** Ottawa, Ontario; 2008.

Additonal Table 1
Sources Retained for Data Extraction and Charting
*** Cited in Review**

143. Craig D, Byrick, R., Carlik, F. : **A physician workforce planning model applied to Canadian anesthesiology: planning the future sup-ply of anesthesiologists.** *Canadian Journal of Anaesthesia* 2002 **49:**671-677.

144. Crutcher RA, Banner, S.R., Szafran, O., Watanabe, M.: **Characteristics of international medical graduates who applied to the CaRMS 2002 match.** *CMAJ: Canadian Medical Association Journal* 2003, **168:**119-1123.

145. Crutcher RA, Tutty, J., Wright, H., Andrew, R., Bourgeois-Law, G., Davis, P., Barron, S., DiNinno, V., Hofmeister, M., Kuehn, N., Mann, Pl, Salte, B., Simkin, R., Violato, C.: **Max Project Report. 'Maximizing the Gains from WAAIP'.** Calgary: Health Sciences Centre University of Calgary; 2007.

146. Crutcher RA, Szafran, O., Woloschuk, W., Chatur, F., Hansesn, C.: **Family medicine graduates' perceptions of intimidation, harassment, and discrimmination during residency training.** *BMC Medical Education* 2011, **11**.

147. Curran V, Hollet, A., Hann, S., Bradbury, C.: **A qualitative study of the international medical graduate and the orientation process.** *Canadian Rural Medicine* 2008, **13:**163-169.

148. *Curran VR, Hollett, A., Allen, M., Steeves, J., Dunbar, P.: **A continuing medical education needs assessment of primary care physicians' knowledge and awareness of prediabetes care.** *Canadian Journal of Diabetes* 2008, **32:**273-280.

149. Cutcliffe JR, Bajkay, R., Forster, S., Small, R., Travale, T.: **Nurse migration in an increasingly interconnected world: The case for internationaliztion of regulation of nurses and nursing regulatory bodies.** *Archives of Psychiatric Nursing* 2011, **25:**320-328.

Additonal Table 1
Sources Retained for Data Extraction and Charting
*** Cited in Review**

150. Dauphinee WD: **Physician migration to and from Canada: the challenge of finding the ethical and political balance between the individual's right to mobility and recruitment to underserved communities.** *Journal of Continuing Education in the Health Professions* 2005, **25:**22-29.

151. *Dauphinee WD: **The circle game: understanding physician migration patterns within Canada.** *Journal of the American Medical Collegers* 2006, **81:**S49-54.

152. Dauphinee WD, Buske, L.: **Medical workforce policy-making in Canada, 1993-2003: reconnecting the disconnected.** *Academic Medicine: Journal of the Association of American Medical Colleges* 2006, **81:**830-836.

153. Davis K: **Responding to the medical laboratory staffing shortage: The Canadian perspective.** *Clinical Leadership & Management Review* 2002, **16:**399-407.

154. de Carvalho M: **The Implications of Being an International Medical Graduate (IMG) in Canadian Society: A Qualitative Study of Foreign-Trained Physicians' Resettlement, Sense of Identify and Health Status.** Brock University, 2007.

155. Deber R: **Internationally educated workers jeopardy: answers and questions.** *Healthcare Papers* 2010, **10:**22-27.

156. Dhalla I, Born, K.: **Why are so many Canadians going abroad to study medicine?** In HealthyDebateca; 2011. http://healthydebate.ca/2011/03/topic/politics-of-health-care/why-are-so-many-canadians-going-abroad-to-study-medicine

157. Dickson KR: **Internationally Educated Health Professionals: Why They Come, Whey They Stay, and the Challenges They Face.** Memorial University of Newfoundland, Psychology; 2007.

158. Dodani S, LaPorte, R.E.: **Brain drain from developing countries: How can brain drain be converted into wisdom gain?** *Journal of the Royal Society of Medicine* 2005, **98**.

Additonal Table 1
Sources Retained for Data Extraction and Charting
*** Cited in Review**

159. Dore KL, Kreuger, S., Ladhani, M., Rolfson, d., Kurtz, D., Kulasegaram, K., Cullimore, AlJ., Norman, G.R., Eva, K. W., Bates, S., Reiter, H. I.: **The reliability and acceptability of the multiple mini-interview as a selection instrument for postgraduate admissions.** *Academic Medicine: Journal of the Association of American Medical Colleges* 2010, **85:**560-563.

160. Dove N: **Can international medical graduates help solve Canada's shortage of rural physicians?** *Canadian Journal of Rural Medicine* 2009, **14:**120-123.

161. Doyle S: **One-stop shopping for international medical graduates.** *CMAJ: Canadian Medical Association Journal* 2010, **182:**1608.

162. Driscoll E: **Administrator calls for global health credentialing.** *CMAJ: Canadian Medical Association Journal* 2009, **180:**E11-E12.

163. Duff P: **Learning language for work and life: The linguistic socialization of immigrant Canadians seeking careers in healthcare.** *THe Modern Language Journal* 2002, **86:**397-422.

164. Dumont J, Zurn, P., Church, J.,: **International Mobility of Health Professionals and Health Workforce Management in Canada: Myths and Realities.** Paris, France: Organisation for Economic Co-operation and Development (OECD); 2008. http://www.oecd.org/canada/41590427.pdf

165. Duncan D, Poisson, Y., Wong, W.: **Improving Bridging Programs: Compiling Best Practices from a Survey of Canadian Bridging Programs.** Ottawa, Ontario; 2008. https://www.ppforum.ca/sites/default/files/bridging_programs%20report_0.pdf

166. Dywili S, Bonner, A,. Anderson, J., O' Brien, L.: **Experience of overseas-trained health professionals in rural and remote areas of destination countries: a literature review.** *Australian Journal of Rural Health* 2012, **20:**175-184.

Additonal Table 1
Sources Retained for Data Extraction and Charting
*** Cited in Review**

167. Elgersma S: **Recognition of the Foreign Qualifications of Immigrants (Background Paper).** Ottawa, Ontario: Parliamentary Information and Research Service; 2012. http://www.lop.parl.gc.ca/content/lop/ResearchPublications/2004-29-e.pdf

168. Emery JCH, Ferrer, A.: **The Social Rate of Return to Investing in Character: An Economic Evaluation of Alberta's Immigrant Access Fund Micro Loan Program.** Calgary, Alberta: University of Calgary; 2010. http://econ.ucalgary.ca/manageprofile//sites/econ.ucalgary.ca.manageprofile/files/unitis/publications/162-65056/EmeryFerrer.pdf

169. Federal/Provincial/Territorial Advisory Committee on Health Delivery and Human Resources: **How Many Are Enough? Redefining Self-Sufficiency for the Health Workforce: A Discussion Paper.** Ottawa, Ontario; 2009. http://www.hc-sc.gc.ca/hcs-sss/pubs/hhrhs/2009-self-sufficiency-autosuffisance/index-eng.php

170. Fleming P, Mathews, M.: **Retention of specialist physicians in Newfoundland and Labrador.** *Open Medicine* 2012, **6**. http://www.openmedicine.ca/article/view/465/444

171. Fooks C, Maslove, L.,: **Policy Initiatives for Physicians, Nurses and Pharmacists: Research Report H07 Health Network.** Ottawa, Ontario; 2004. . http://books1.scholarsportal.info/viewdoc.html?id=25455

172. Foster L: **Foreign trained doctors in Canada: Cultural contingency and cultural democracy in the medical profession.** *International Journal of Criminology and Sociological Theory* 2008, **1:**1-25.

173. Frank B, Saunders, P.: **Integration of International Medical Graduates in Rural Nova Scotia Communities. A Qualitative Pilot Study.** vol. Working Paper No. 23. Halifax, Nova Scotia, Canada; 2008. http://community.smu.ca/atlantic/documents/WP23FrankSaunders.pdf

Additonal Table 1
Sources Retained for Data Extraction and Charting
*** Cited in Review**

174. Gallant G: **Web-Based Mentorship Module to Facilitate Internationally Educated Nurses' Transition into Practice in Canada: Early Results.** In *14 International Conference on Interactive Collaborative Learning (ICL2011)-11th International Conference University*. pp. 519-524. Slovakia; 2011:519-524.

175. Garibaldi RA, Subhlyah, R., Moore, M. E., Waxman, H.: **The in-training examination in internal medicine: an analysis of resident performance over time.** *Annals of Internal Medicne* 2002, **137:**505-510.

176. Glover Takahashi S, McIlroy, J., Beggs, C., SGT & Associates,: **Assessing the Competence of Internationally Educated Occupational Therapists for Practise in Canada. Towards a Common Approach and an Assessment Toolkit.** Victoria, British Columbia: College of Occupational Therapists of British Columbia; 2008. http://www.acotro-acore.org/sites/default/files/uploads/acotro_ieot_phase_1_report_may_31_2008.pdf

177. Glover Takahashi S, McIlroy, J., Beggs, C. : **Assessing Substantial Equivalence of Internationally Educated Occupational Therapists: Principles, Process & Tools.** Victoria, British Columbia: College of Occupational Therapists of British Columbia; 2011. http://www.acotro-acore.org/sites/default/files/uploads/se_2011_webversion.pdf

178. Goldszmidt M, Kortas, C., Meehan, S.: **Advanced medical communications: support for international residents.** *Medical Education* 2007, **41:**522.

179. Government of Alberta: **Health Workforce Action Plan.** Edmonton, Alberta; 2006. http://www.health.alberta.ca/documents/Workforce-Action-Plan-2007.pdf

180. Government of Nova Scotia: **Welcome Home to Nova Scotia. A strategy for Immigration.** Nova Scotia, Canada: Government of Nova Scotia; 2005. http://0-nsleg-edeposit.gov.ns.ca.legcat.gov.ns.ca/deposit/b10627340.pdf

Additonal Table 1
Sources Retained for Data Extraction and Charting
*** Cited in Review**

181. Government of Ontario: **Progress Report 2005. Working Together for a Better Ontario.** Ontario, Canada; 2005.

182. *Grant H: **From the Transvaal to the Prairies: The Migration of South African Physicians Canada.** *Journal of Ethnic and Migration Studies* 2006, **32:**681-695.

183. Grant M: **Internationally-educated medical laboratory technologists: the story of one or many?** *Canadian Journal of Medical Laboratory Sciences* 2008, **70:**133-135.

184. Grant M: **Fair and valid standards for language proficiency testing: CSMLS leads the way.** *Canadian Journal of Medical Laboratory Sciences* 2009, **71:**189-190.

185. Grant M: **Bridging Programs for Internationally Educated Medical Laboratory Technologists. A Business Case. Final Report.** Hamilton, Ontario, Canada; 2009. https://csmls.org/csmls/media/documents/publications/reports/BridgingPrograms_FinalReport_EN.pdf

186. *Grant M: **The CSMLS task torce on internationally educated medical laboratory technologists: an overview** *Canadian Journal of Medical Laboratory Sciences* 2009, **71:**129-131.

187. Grant M: **Final Project Report, Outcomes Indicators and Tracking Mechanisms. Addressing The Competency Gaps of Internationally Educated Medical Laboratory Technologists.** Hamilton, Ontario; 2010. https://csmls.org/csmls/media/documents/publications/reports/Competency_Gaps_Final_Report.pdf

188. *Grant M: **CSMLS members' perspectives on internationally educated medical laboratory technologists and human resources.** *Canadian Journal of Medical Laboratory Sciences* 2010, **72:**134-143.

Additonal Table 1
Sources Retained for Data Extraction and Charting
*** Cited in Review**

189. Grant M: **Loan Libraries: Supporting the Professional Preparation of Internationally Educated Medical Laboratory Technologists.** Hamilton, Ontario, Canada: Canadian Society for Medical Laboratory Scinece; 2010. https://csmls.org/csmls/media/documents/publications/reports/LOAN_LIBRARIES_-_FINAL_external_report.pdf

190. Grant M: **Subject certification: An analysis of CSMLS members views.** *Canadian Journal of Medical Laboratory Sciences* 2011, **73:**25-33.

191. *Grant M, Strachan AL, Neilsen C, Verburg M: **Investigation of Language Assessment Tools and Benchmarks Necessary for Success for Internationally Educated Medical Laboratory Technologists.** In *Canadian Journal of Medical Laboratory Sciences*. Hamilton, Ontario; 2008. https://csmls.org/csmls/media/documents/publications/reports/csmls_final_report_-_investigation_of_language_tools.pdf

192. Gregory J: **Internationally Educated Nurses Experience Survey Review.** Saskatchewan; 2011.

193. Griffiths H: **So long home, hello Canada: foreign-educated nurses.** *Nursing BC* 2001, **33:**16.

194. Gushue J: **Newfoundland seeks solution to MD turnover.** *Canadian Medical Association Journal* 2000, **163:**434.

195. Hagley R, Choudhry, U., Guruge, S., Turrittin, J., Collins, E., Lee, R.: **Immigrant nurses' experience of racism.** *Journal of Nursing Scholarship* 2001, **33:**389-394.

196. Haley B, Simosko, S.: **Prior Learning Assessment and Internationally Trained Medical Laboratory Technologists: Capstone Report.** Hamilton, Ontario; 2011.

Additonal Table 1
Sources Retained for Data Extraction and Charting
*** Cited in Review**

197. Hall P, Keely, E., Dojeiji, S., Byszewski, A., Marks, M.: **Communication skills, cultural challenges and individual support: challenges of international medical graduates in a Canadian healthcare environment.** *Medical Teacher* 2004, **26:**120-125.

198. Hamilton C: **Successful integration of IENs into the workplace... internationally educated nurses.** *SRNA Newsbulletin* 2008, **10:**27.

199. *Hamilton C: **Capacity building for internationally educated nurses assessment project.** *SRNA Newsbulletin* 2009, **11**.

200. Health Canada: **Health Human Resources: Balancing Supply and Demand.** *Health Policy Research Bulletin* 2004. . http://www.hc-sc.gc.ca/sr-sr/pubs/hpr-rpms/bull/2004-8-hhr-rhs/index-eng.php

201. Health Canada (HC): **Health Human Resource Strategy and Internationally Educated Health Professionals Initiative - 2009-2010 Annual Report.** Ottawa, Ontario; 2010.

202. HealthForceOntario: **Ontario's New Physician Recruitment and Retention Programs Bringing the Underserviced Area Program Into the 21st Century. A Consultation Paper.**, vol. Spring/Summer 2009. Ottawa, Ontario: HealthForceOntario; 2009.

203. *HealthForce Ontario. **Access Centre** [http://www.healthforceontario.ca/en/M4/Internationally_Educated_Health_Professionals]

204. Hearnden MV: **Nursing Across Cultures: The Communicative Needs of Internationally Educated Nurses Working with Older Adults.** Ontario institute for Studies in Education, University of Toronto, Curriculum, Teaching and Learning; 2007.

Additonal Table 1
Sources Retained for Data Extraction and Charting
*** Cited in Review**

205. Hefley J, Mandel,J., Gerace, R. : **Internationally educated healthcare workers: Focus on physicians in Ontario. A commentary.** *Healthcare Papers* 2010, **10:**41-45.

206. Hewitt Associates: **Assessment and Recommendations for Attracting and Retaining Registered Nurses.** Newfoundland and labrador; 2010. *http://www.health.gov.nl.ca/health/publications/assessment_and_recommendations.pdf*

207. Higginbottom GM: **The transitioning experiences of internationally-educated nurses into a Canadian health care system: A focused ethnography.** *BMC Nursing* 2011, **10**.

208. Higginbottom GMA: **Experience and Transitioning of Internationally Educated Nurses (IENS) into the Alberta Healthcare System** In *The PMC Working Paper Series is published by the Prairie Metropolis Centre* 2010.

209. Hoag H: **Canada increasingly reliant on foreign-trained health professionals.** *CMAJ : Canadian Medical Association Journal* 2008, **178:**270.

210. Hofmeister M, Lockyer, J., Crutcher, R.: **The multiple mini-interview for selection of international medical graduates into family medicine residency education.** *Medical Education* 2009, **43** 573-579.

211. *Human Resources and Skills Development Canada: **Summative Evaluation of the Foreign Credential Recognition Program.** (Branch EDSPaR ed. Gatineau, Quebec, Canada; 2010.

212. Humber N, Frecker, T.: **Delivery models of rural surgical services in British Columbia (1996-2005): Are general practitioner-surgeons still part of the picture?** *Canadian Journal of Surgery* 2008, **51:**173-178.

Additonal Table 1
Sources Retained for Data Extraction and Charting
*** Cited in Review**

213. Isreal N: **Recognition of Prior Learning in Regulated Professions: Environmental Scan.** In *Data Point*. Ontario, Canada; 2011.

214. *Jablonski JOD: **Employment Status and Professional Integration Outcomes of IMGs in Ontario.** Epidemology; 2012.

215. Jacob B, Baxter, N.N., Moineddin, R., Sutradhar, R., Del Giudice, L., Urbach, D.R.: **Social disparities in the use of colonoscopy by primary care physicians in Ontario.** *BMC Gastroenterology* 2011, **11**.

216. Jain G, Mazhar, M.N., Uga, A., Punwani, M., Broquet, K.E.: **Systems-based aspects in the training of IMG or previously trained residents: Comparison of psychiatry residency training in the United States, Canada, theUnited Kingdom, India, and Nigeria.** *Academic Psychiatry* 2012, **36:**307-315.

217. Jeans M, Hadley, F., Green, J.,: **Navigating To Become A Nurse In Canada: Assessment of International Nurse Applicants: Final Report.** Ottawa, Ontario; 2005. . https://www.cna-aiic.ca/~/media/cna/page-content/pdf-fr/ien_technical_report_e.pdf?la=en

218. Jeans ME: **In-country challenges to addressing the effects of emerging global nurse migration on health care delivery** *Policy, Politics & Nursing Practice* 2006, **7:**58S -61S. https://www.cna-aiic.ca/~/media/cna/page-content/pdf-fr/ien_technical_report_e.pdf?la=en

219. Johnson K: **Integrating internationally educated physiotherapists.** Canadian Alliance of Physiotherapy Regulators & Canadian Physiotherapy Association; 2007. . www.alliancept.org/pdfs/cred_iiep_integrate_eng.pdf.

Additonal Table 1
Sources Retained for Data Extraction and Charting
*** Cited in Review**

220. Johnson K, Israel, N.: **Towards a Pan-Canadian Bridging Program for International Applicants.** Montreal, Quebec; 2011. https://www.google.ca/#q=Towards+a+Pan-Canadian+Bridging+Program+for+International+Applicants.+Final+Report.+Canadian+Alliance+of+Respiratory+Therapists+Regulatory+Bodies.+Montreal%2C+Quebec.

221. *Johnson K, Baumal B: **Assessing the Workforce Integration of Internationally Educated Health Professionals: Final Report.** 2011. https://www.caot.ca/pdfs/WFI_Report_E.pdf

222. Johnston S: **Quality not quantity: What do we want for the future?** *Journal of Obstetrics and Gynaecology Canada* 2007, **29:**495-496.

223. *Joudrey RJR, K.: **Practising medicine in two countries: South African physicians in Canada.** *Sociology of Health & Illness* 2010, **32:**528-544.

224. Kabene SM, Howard, J.M., Zhou, N.X.: **Addressing the physician shortage with international medical graduates: the current situation and solutions.** *The Internet Journal of Healthcare Administration* 2009, **6**.

225. Katikireddi V: **Canada and UK must stop taking African doctors.** *CMAJ: Canadian Medical Association Journal* 2005, **173:**584.

226. Kawi J, Xu, Y.: **Facilitators and barriers to adjustment of international nurses: an integrative review.** *International Nursing Review* 2009, **56:**174-183.

227. Keatings M: **Health services delivery: reframing policies for global nursing migration to North America- A Canadian perspective.** *Policy, Politics, & Nursing Practice* 2006, **7:**62S-65S.

Additonal Table 1
Sources Retained for Data Extraction and Charting
*** Cited in Review**

228. Keenan M: **To Ireland and back.** *Canadian Family Physician* 2005, **51:**1431-1432.

229. Khaliq AA, Broyles, R. W., Mwachofi, A. K.,: **Global nurse migration: its impact on developing countries and prospects for the future.** *Nursing Leadership* 2009, **22:**24-50.

230. Khan K, Campbell, A., Wallington, T., Gardam, M.: **The impact of physician training and experience on the survival of patients and active tuberculosis.** *CMAJ: Canadian Medical Association Journal* 2006, **175:**749-753.

231. Klein HH, M,.; Lockyer, J.; Crutcher, R.; Fidler, H.: **Push, pull, and plant: The personal pride of physician immigration to Alberta, Canada.** *International Family Medicine* 2009, **41:**197-201.

232. Kline DS: **Push and pull factors in interntional migration.** *Journal of Nursing Scholarship* 2003, **35:**107-1011.

233. Ko DT, Austin, P.C., Chan, B.T.B., Tu, J.V.: **Quality of care of international and Canadian medical graduates in acute myocardial infarction.** *Archives of Internal Medicine* 2005, **165:**458-463.

234. *Kogo S: **Migration of African-Trained Physicians Abroad: A Case Study of Saskatchewan, Canada.** University of Saskatchewan, Geography; 2009.

235. Kogon SB, D.; Sandhu, H.,: **Foreign-trained dentists' perceived knowledge and skills after graduation from a structured two-year program.** *Journal of Dental Education* 2011, **75:**1098-1106.

236. Kolawole B: **Ontario's internationally educated nurses and waste in human capital: Opinion Piece of International Interest.** *International Nursing Review* 2009, **56:**184-190.

Additonal Table 1
Sources Retained for Data Extraction and Charting
*** Cited in Review**

237. Kolawole B: **International nurse migration to Canada: Are we missing the bigger picture?** *Nursing Leadership* 2010, **23:**16-20.

238. Komarnicki E: **A Framework for Success: Practical Recommendations to Further Shorten the Foreign Qualifications Recognition Process. Report of the Standing Committee on Human Resources, Skills and Social Development and the Status of Persons with Disabilities.** Ottawa, Ontario: House of Commons Canada; 2012.

239. Kondro W: **Credentialing body needed for foreign-trained doctors.** *CMAJ: Canadian Medical Association Journal* 2002, **171:**435.

240. Kondro W: **ONE MATCH fits all?** *Canadian Medical Association Journal* 2006, **175:**138-139.

241. Kondro W: **National resident match emerges for IMGs.** *Canadian Medical Association Journal* 2006, **175:**236.

242. Kondro W: **Alternative remuneration.** *CMAJ: Canadian Medical Association Journal* 2007, **176:**1573.

243. Kondro W: **Trends in the profile of the Canadian physician pool.** *CMAJ: Canadian Medical Association Journal* 2009, **180:**284.

244. Kroeker D: **A Resource Guide for Internationally Educated Registered Nurses: Information to Help You Plan Your Career in Manitoba.** Manitoba, Canada; 2008. http://www.healthadvocates.info/IMG/articles_litreview/ManitobaResourceGuide.pdf

245. Labonte R, Packer, C., Klassen, N.: **Managing health professional migration from sub-Saharan Africa to Canada: A stakeholder inquiry into policy options.** *Human Resources for Health* 2006, **4**.

Additonal Table 1
Sources Retained for Data Extraction and Charting
*** Cited in Review**

246. *Lafontant J, Forgues, E., Belkhodja, C., Sangwa-Lugoma, G., Meridji, T., Pietrantonio, L., Tremblay, A., Kayirangwa, C.: **La Reconnaissance des Diplômes Internationaux Francophones en Santé : Un Potentiel Pour les Communautés Francophones en Situation Minoritaire au Canada.** Montreal, Quebec; 2006.

247. Landry MD, Gupta N, Tepper J: **Internationally educated health professionals and the challenge of workforce distribution.** *Healthcare Papers* 2010, **10:**35-40.

248. *Lax L, Lynn Russell M, Nelles LJ, Smith CM: S**caffolding knowledge building in web-based communication and cultural competence program for inernational medical graduates.** Journal of the Association of American Medical Colleges 2009, **10**: 35-40.

249. Lindberg M: **Internationally educated nurses in Saskatchewan: it's not easy but it's worth it!** In *SRNA Newsbulletin*, vol. 10: Saskatchewan Registered Nurses Association (SRNA); 2008. http://www.srna.org/images/stories/pdfs/communications/newsbulletin/bulletin_apr_08.pdf.

250. Little L: **Nurse migration: A Canadian case study.** *Health Services Research* 2007, **42**.

251. Lockyer J, Blackmore, D., Fidler, H., Crutcher, R., Salte, B., Shaw, K., Ward, B., Wolfish, N.: **A study of a multi-source feedback system for international medical graduates holding defined licenses.** *Medical Education* 2006, **40:**340-347.

252. *Lockyer J, Hofmeister, M., Crutcher, R., Klein, D., Fidler, H.: **International medical graduates: Learning for practice in Alberta, Canada.** *Journal of Continuing Education in the Health Professions* 2007, **27:**157-163.

253. Lockyer J, Fidler, A., De Gara, C., Keefe, J.: **Mentorship for the physician recruited from abroad to Canada for rural pracice.** *Medical Teacher* 2010, **32:**e322-e327.

Additonal Table 1
Sources Retained for Data Extraction and Charting
*** Cited in Review**

254. Loewen S, Brundage, M., Tankel, K., Fairchild, A., Trotter, T., Wiebe, E., Ingledew, P.A., Stuckless, T., Yee, D.: **Radiation oncology workforce recruitment survey of 2000-2010 graduates: Is there a need for better physician resource planning?** *Canadian Medical Education Journal* 2012, **3:**e52-263.

255. Lombard AC: **Retention of Health Professionals in Rural Nova Scotia.** Halifax, Nova Scotia: Dalhousie University; 2005.

256. Lum L: **Accommodating Learning Styles In Bridging Education Programs For Internationally Educated Professionals (IEPs).** Toronto, Ontario: York University; 2009.

257. Maamoun J: **The need for a transitional program for internationally educated radiation therapists.** *Canadian Journal of Medical Radiation Technology* 2007, **38:**14-19.

258. MacDonald-Rencz S, Davies, J.: **The health system and international recruitment.** *Healthcare Papers* 2010, **10:**28-34.

259. MacLean C: **Turf wars? .** *Canadian Family Physician* 2010, **56:**297-298.

260. MacLellan A, Brailovsky, C., Miller, F., Leboeuf, S.: **Clerkship pathway: A factor in certification success for international medical graduates.** *Canadian Family Physician* 2012, **58:**662-227.

261. MacLellan AM, Brailovsky, C., Rainsberry, P., Bowmer, I., Desrochers, M.: **Examination outcomes for international medical graduates pursuing or completing family medicine residency training in Quebec.** *Canadian Family Physician* 2010, **56:**912-918.

Additonal Table 1
Sources Retained for Data Extraction and Charting
*** Cited in Review**

262. MacPherson G: **Examining an Orientation Progarm for International Medical Graduates (IMGs) Through the Lens of Critical Theory: A Learner-Centred Program.** Mount Saint vincent University, 2011. http://ec.msvu.ca:8080/xmlui/bitstream/handle/10587/1132/GwenMacPhersonMAEdThesis2011.pdf?sequence=1

263. Magnus B: **Foreign-trained doctors dominate pilot project.** *CMAJ: Canadian Medical Association Journal* 2008, **178:**1411.

264. Mahamed A, Gregory, P.A.M., Austin, A.: **"Testwiseness" among international pharmacy graduates and Canadian senior students.** *American Journal of Pharmaceutical Education* 2006, **70:**1-6.

265. Management Committee: **Moving Forward: Pharmacy Human Resources for the Future: Final Report.** Ottawa, Ontario; 2008. http://blueprintforpharmacy.ca/docs/default-document-library/2011/04/19/Moving%20Forward%20Final%20Report.pdf?Status=Master

266. Manitoba Regional Health Authority External Review Committee: **Health Best Practices.** Winnipeg, MB, Canada; 2008.

267. Marchildon G, O'Fee, K.: **Health Care Saskatchewan: An Analytical Profile.** Regina, SK, Canada: Canadian Plains Research Centre; 2008.

268. Martin W, Morgan, C.: **Analysis of the International Questionnaire Regarding the Evaluation of Foreign-Educated Professionals.** Ottawa, Ontario; 2005.

269. Masalmeh S: **Integrating International Medical Graduates: Nova Scotia Resources and Gaps.** Dalhousie University, Health Administration; 2009.

Additonal Table 1
Sources Retained for Data Extraction and Charting
*** Cited in Review**

270. Matejicek AM: **(Re)Constructing the Meaning of Work: Experience of Internationally Trained Female Physicians Who Immigrate to Canada.** The University of Guelph, 2008.

271. Mathews M, Park, A., Rourke, J.T.: **Retention of international medical graduates following postgraduate medical training in Newfoundland and Labrador.** *Healthcare Papers* 2007, **3:**50-58.

272. Mathews M, Edwards, A.C., Rourke, J. T.: **Retention of provisionally licensed international medical graduates: a historical cohort study of general and family physicians in Newfoundland and Labrador.** *Open Medicine* 2008, **2:**e62-69.

273. Mathews M, Rourke, J. T.B., Park, A.: **National and provincial retention of medical graduates of Memorial University of Newfoundland.** *CMAJ: Canadian Medical Association Journal* 2009, **175:**357-360.

274. Maudsley RF: **Assessment of international medical graduates and their integration into family practice: the clinician assessment for practice program.** *Academic Medicine: Journal of the Association of American Medical Colleges* 2008, **83:**309-315.

275. Mayo E, Mathews, M. : **Spousal perspectives on factors influencing recruitment and retention of rural family physicians.** *Canadian Journal of Rural Medicine* 2006, **11:**271-276.

276. McDonald JT, Worswick, C.: **The determinants of migration decisions of immigrant and non-immigrant physicians in Canada.** Hamilton, Ontario, Canada: McMaster University; 2010. http://socserv.mcmaster.ca/sedap/p/sedap282.pdf

277. McDonald JT, Warman, C., Worswick, C.: **Earnings, occupation, and schooling decisions of immigrants with medical degrees: Evidence for Canada and the United States.** In *High-Skilled Immigration in a Globalized Labor Market: Research in Labor Economics.* Washington, D.C: American Enterprise Institute for Public Policy Research; 2010

Additonal Table 1
Sources Retained for Data Extraction and Charting
*** Cited in Review**

278. McGrath P, Wong, A., Holewa, H.: **Canadian and Australian Licensing Policies for International Medical Graduates: A Web-based Comparison.** In *Education for Health*, vol. 1: 24; 2011. www.educationforhealth.net.

279. McGuire M, Murphy,S.: **The internationally educated nurse: Well-researched and sustainable programs are needed to introduce internationally educated nurses to the culture of nursing in Canada.** *Canadian Nurse* 2005, **101:**25-29.

280. McIntosh T: **IEHPs, Sustainability and Ethics: The Need for a Pan-Canadian HHR Grow-Op.** In *International Symposium on Health Innovation*. Vancouver, British Columbia: Canadian Policy Research Networks; 2007.

281. *McIntosh T, Torgerson R, Klassen N: **The Ethical Recruitment of Internationally Educated Health Professionals: Lessons from Abroad and Options for Canada.** Ottawa, Ontario; 2007. http://rcrpp.ca/documents/46781_en.pdf

282. McKenna M, Ganbesan, S., Soma, R.: **Academic and educational role of international medical graduate psychiatrists in British Columbia.** *BC Medical Journal* 2007, **49**.

283. *McMahon S: **Preliminary Report of Canadian Models for IMG Competency Assessment** In *Creating a Fair and Equitable Fast Track Assessment Process for International Medical Graduates* ([AIMGA] AIMG ed. Ottawa, Ontario, Canada; 2009.

284. Med-Emerg Inc.: **Phase II Final Report.** In *Building the Future: An Ingtegrated Strategy for Nursing Human Resources in Canada*. Ottawa, Ontario; 2006. https://nursesunions.ca/sites/default/files/PHASE_II_FINAL.pdf

285. Medical Council of Canada: **Recalibrating for the 21st Century. Report of the Assessment Review Task Force of the Medical Council of Canada.** Ottawa, Ontario; 2011. http://mcc.ca/wp-content/uploads/Reports-assessment-review-task-force.pdf

Additonal Table 1
Sources Retained for Data Extraction and Charting
*** Cited in Review**

286. Medical Council of Canada: **Medical Council of Canada Evaluating Examination** [http://mcc.ca/examinations/]

287. Meslay A, Bessard, M.J. , Rivera. L.C., Chesa, M. M.: **[Welcome. Immigrant nurses. Interview by Jean-Marc Papineau].** *Perspective Infirmiere* 2008, **5:**6-9.

288. Miller PA, Cooper, M.A., Eva, K.W.: **Factors predicting competence as assessed with the written component of the Canadian Physiotherapy Competency Examination.** *Physiotherapy Theory and Practice* 2010, **26:**12-21.

289. *Mills EJ, Schabas WA, Volmink J, Walker R, Ford N, Katabira E, Anema A, Joffres M, Cahn P, Montaner J: **Should active recruitment of health workers from sub-Saharan Africa be viewed as a crime?** *Lancet* 2008, **371:**685-688.

290. Ministry of Health and Long Term Care: **Guidelines for Return of Service (ROS) for International Medical Graduates (IMGs), Repatriation Program and Registration through Practice Assessment.** Ontario, Canada: HEALTH HUMAN RESOURCES POLICY BRANCH 2007.

291. Minore B, Pong, R., Ariss, R.: **A Situational Analysis of Physician Recruitment and Retention n Rural and Northern Canada: Models, Programs and Evaluations. Final Report.** Thunder Bay & Sudbury, Ontario: Lakehead University and Laurentian University; 2001.

292. Mok PS, Baerlocher, M.O., Abrahams, C., Tan, E.Y., Slade, S., Verma, S. : **Comparison of Canadian medical graduates and international medical graduates in Canada: 1989-2007.** *Academic Medicine: Journal of the Association of American Medical Colleges* 2011, **86:**962-967.

293. Mont Royal University: **Professional Communication for Internationally Educated Health Professionals Project (PC-IEHP) An Alberta Pilot for Professional Communication.** Calgary, Alberta: Mont Royal University; 2011. http://www.mtroyal.ca/cs/groups/public/documents/pdf/pciehp_public_report_2011.pdf

Additonal Table 1
Sources Retained for Data Extraction and Charting
*** Cited in Review**

294. Muhammad Gadit AA: **International migration of doctors from developing countries: need to follow the Commonwealth Code.** *Journal of Medical Ethics* 2008, **34:**67-68.

295. Mullan F: **The metrics of physician brain drain.** *The New England Journal of Medicine* 2005, **353:** 1810-1818.

296. Murphy S: **Actualizing the Dream: experiences of internationally-educated nurses.** University of Calgary (Canada), 2008.

297. Nasmith L: **License requirements for international medical graduates: should national standards be adopted?** *CMAJ: Canadian Medical Association Journal* 2000, **162:**795-796.

298. National Dental Assisting Examination Board [NDAEB]: **Annual Report.** Ottawa, Ontario; 2011. http://www.ndeb.ca/nonaccredited.

299. National Dental Assisting Examination Board [NDAEB]: **Graduates of Non-Accredited Dental Programs** [http://www.ndeb.ca/nonaccredited]

300. Neilsen C: **Assessing the workforce integration of internationally educated health professionals.** *Canadian Journal of Medical Laboratory Sciences* 2010, **72:**73.

301. Neiterman E, Bourgeault, IL: **Conceptualizing professional diaspora: International medical graduates in Canada.** *International Migration and Integration* 2012.

302. Nelson LJ: **The transition to practice in Canada: The experiences of nurses educated outside of Canada.** University of British Columbia, nursing; 2005.

Additonal Table 1
Sources Retained for Data Extraction and Charting
*** Cited in Review**

303. Nelson S, Verma, S., Hall, L., Gastalda, D., Janjua, M.: **The shifting landscape of immigration policy in Canada: Implications for health human resources.** *Health Policy* 2011, **7:**60-67.

304. Newton S, Pillay, J., Higginbottom,G.: **The migration and transitioning experiences of internationally educated nurses: a global perspective.** *Journal of Nursing Management* 2012, **20:**534-550.

305. Nguyen T, Baptiste, S.: **The McMaster Assessment of Comprehension and Communication: Facilitating the acculturation of internationally educated occupational therapists.** *Occupational Therapy Now* 2011, **13:**19-20.

306. Nousiainen M, Latter, D.A., Backstein, D., Webster, F., Harris, K.A.: **Surgical fellowship training in Canada: What is its current status and is improvement required?** *Canadian Journal of Surgery* 2012, **55:**58-65.

307. O'Brien-Pallas L, Wang, S.: **Innovations in health care delivery: Responses to global nurse migration--A research example.** *Policy, Politics, & Nursing Practice* 2006, **7**.

308. O'Meara D: **Foreign-trained physicians need residency spots.** *CMAJ: Canadian Medical Assocation Journal* 2004, **170:**1216.

309. Office of the Fairness Commissioner: **Ontario's Regulated Professions: Report on the 2007 Study of Registration Practices.** In *Office of the Fairness Commissioner*. Toronto, Ontario; 2008. http://www.fairnesscommissioner.ca/index_en.php?page=about/media_room/news_release_080626

310. Office of the Fairness Commissioner: **Study of Qualifications Assessment Agencies.** Toronto, Ontario; 2009. http://www.fairnesscommissioner.ca/files_docs/content/pdf/en/study_of_qualifications_assessment_agencies_print_pdf_english.pdf

Additonal Table 1
Sources Retained for Data Extraction and Charting
*** Cited in Review**

311. Office of the Fairness Commissioner: **Entry-to-Practice Requirements for Five Professions in Five Canadian Provinces.** Toronto, Ontario; 2010. . http://www.fairnesscommissioner.ca/files_docs/content/pdf/en/Entry-to-Practice_Requirements.pdf
312. Office of the Fairness Commissioner: **Getting Your Professional Licence in Ontario: The Experience of International and Canadian Applicants.** (LTd. RAMA ed. Toronto, Ontario; 2010. http://www.fairnesscommissioner.ca/files_docs/content/pdf/en/Getting_Your_Professional_Licence_in_Ontario-The_Experiences_of_International_and_Canadian_Applicants.pdf
313. Official Languages Community Development Bureau (OLCDB): **Overview of the Cost of Training Health Professionals.** Ottawa, Ontario: Health Canada,; 2008.
314. Ogilvie L, Long, B.: **Licensure of internationally educated nurses seeking professional careers in the province of Alberta in Canada.** *International Migration and Integration* 2007, **8:**223-241.
315. Ogilvie L, Mill, J.E., Astle, B., Fanning, A., Opare, M.: **The exodus of health professionals from sub-Saharan Africia: balancing human rights an societal needs in the twenty-first centrury.** *Nurisng Inquiry* 2007, **14:**112-124.
316. Ontario Ministry of Health and Long Term Care: **How to Become a Doctor in Ontario: Information for International Medical Graduates. Opportunities and Challenges for International Medical Graduates.** Toronto, Ontario, Canada; 2005. http://www.davidgargaro.com/portfolio/BecomeDoctor.pdf
317. Ordre des infirmières et infirmiers du Québec (OIIQ): **Les Infirmières Formées a L'étranger et l'Acces a la Profession au Quebec.** Ordre des infirmières et infirmiers du Quebec (OIIQ); 2004. http://www.oiiq.org/uploads/publications/memoires/acces_profession.pdf

Additonal Table 1
Sources Retained for Data Extraction and Charting
*** Cited in Review**

318. Ordre des infirmières et infirmiers du Québec (OIIQ): **L'Intégration Professionnelle des Infirmières Immigrantes. Memoire.** Montreal, Quebec; 2007. http://www.oiiq.org/publications/repertoire/lintegration-professionnelle-des-infirmieres-immigrantes

319. *Ordre des infirmières et infirmiers du Québec (OIIQ): **How to Obtain a Nursing Permit from the Ordre des Infirmieres et Infirmiers du Quebec.** pp. 10. Montreal, Quebec; 2012:10. https://www.oiiq.org/sites/default/files/uploads/pdf/publications/publicationsoiiq/Supplement-anglais.pdf

320. Osmond B: **Policy Barriers to Recruitment and Retention of Health Professionals in Rural Areas of Nova Scotia. Summary Report.** Halifax, Nova Scotia; 2004. http://www.ruralnovascotia.ca/documents/rural%20health/recruit%20retention%20summary%20report%202004.pdf

321. Pereira A, Shinewald, B., Wise, A., Yates, S., Young, R.: **Moving in the Right Direction? Labour Mobility, Labour Shortage and Canada's Human Potential.** Vancouver, British Columba; 2007. http://www.actioncanada.ca/wp-content/uploads/2014/04/moving-in-the-right-direction-0607.pdf

322. Peters C: **The Bridging Education and LIcensure of International Medical Doctors in Ontario: A Call for Commitment, Consistency, and Transparency.** University of Toronto, Ontario Institute for Studies in Education; 2011.

323. Phillip RM: **The IEN journey.** *Alberta RN* 2008, **64:**16.

324. Pittman P, Herrera,C., Spetz,J., Davis,C. R.: **Immigration and contract problems experienced by foreign-educated nurses.** *Medical Care Research & Review* 2012, **69:**351-356.

Additonal Table 1
Sources Retained for Data Extraction and Charting
*** Cited in Review**

325. Prince Edward Island- ANC: **Microcredit Report-Development of a Microcredit Model for IEHPs Living in Atlantic Canada.** Prince Edward Island, Canada; 2011.

326. Progress Centre Planning Institute (PCPI): **Winning Strategies for IEHPs' Success in the Workplace: Employers' and IEPs' Perceptions.** Toronto, Ontario, Canada: Progress Career Planning Institute; 2011. https://www.iep.ca/11documents/2011_IEP_Research_Study_Eng.pdf

327. Province of Nova Scotia: **Shaping Our Physician Workforce.** Halifax, Nova Scotia; 2012. http://novascotia.ca/dhw/publications/Physician_Resource_Plan_Shaping_our_physician_Workforce.pdf

328. Province of Saskatchewan: **11-12 Annual Report. Physician Recruitment Agency of Saskatchewan.** Saskatoon, Saskatchewan; 2012. http://www.finance.gov.sk.ca/PlanningAndReporting/2011-12/201112HealthAnnualReport.pdf

329. Pylypa J: **Portrayals of global health worker migration in Canadian print news media: Domestic concerns vs. global awareness.** *International Migration and Integration* 2011.

330. *R. A. Malatest & Associates Ltd R, M.: **Integration of International Pharmacy Graduates into the Canadian Pharmacy Workforce: Barriers and Facilitators.** In *Moving Forward Pharmacy Human Resources for the Future*. Ottawa, Ontario; 2008. http://docplayer.net/8339059-Integration-of-international-pharmacy-graduates-into-the-canadian-pharmacy-workforce-barriers-and-facilitators.html

331. Rao NR: **Psychodynamic psychotherapy training as acculturative experience for international medical graduates: a commentary.** *Academic Psychiatry* 2012, **36:**271-276.

Additonal Table 1
Sources Retained for Data Extraction and Charting
*** Cited in Review**

332. Registered Nurses Association of Alberta: **The challenge of getting internationally educated nurses to the frontline.** *Alberta RN* 2008, **64:**10.

333. Registered Nurses Association of Alberta: **Learning from experience project: Improving the process of internationally educated nurses' applications for registration.** *Alberta RN* 2012, **68:**6.

334. Registered Nurses Association of Ontario (RNAO): **Recruitment of Internationally Educated Nurses.** Toronto, Ontario; 2008. http://rnao.ca/policy/reports/Recruitment-Internationally-Education-Nurses-Policy-Brief

335. Registered Nurses of Nova Scotia: **Challenges faced by IENs. Call for Innovative New Strategies. 2nd part in a three part series.** *Nursing in Focus* 2010, **11:**13-15.

336. Ridewood S: **IENs bridge learning curve with challenge and support.** *Alberta RN* 2010, **66:**13-15.

337. Ronquillo C: **Immigrant Filipino Nurses in Western Canada: An Exploration of Motivation and Migration Experiences through Oral History.** University of British Columbia, Master of Science in Nursing; 2007.

338. Ronquillo C, Boschma,G., Wong, S. T., Quiney,L.: **Beyond greener pastures: exploring contexts surrounding Filipino nurse migration in Canada through oral history.** *Nursing Inquiry* 2011, **18:**262-275.

339. Ronquillo C: **Leaving the Philippines: oral histories of nurses' transition to Canadian nursing practice.** *Canadian Journal of Nursing Research* 2012, **44:**96-115.

340. Rosen R: **Filipino Nurses in Canada.** vol. 4: Canadian Womens Health Network; 2001. http://www.cwhn.ca/en/node/39594.

Additonal Table 1
Sources Retained for Data Extraction and Charting
*** Cited in Review**

341. Rothman AI, Cusimano, M.: **A comparison of physician examiners', standardized patients' and communication experts' ratings of international medical graduates' english proficincy.** *Academic Medicine: Journal of the Association of American Medical Colleges* 2000, **75:**1206-1211.

342. Rothman AI, Cusimano, M.: **Assessment of English proficiency in international medical graduates by physician examiners and standarized patients.** *Medical Education* 2001, **35:**762-766.

343. Royal College of Physicians and Surgeons of Canada (RCPSC): **RCPSC Statement on Appropriate Physician Resources for Canada: Toward Achieving Responsible Self-Sufficiency.** vol. Resolution No. 2005-090. Ontario, Canada: Royal College of Physicians and Surgeons of Canada; 2006.

344. Royal College of Physicians and Surgeons of Canada (RCPSC): **One Step ForwardTwo Steps Back? A discussion Paper on Physician Mobility in Canada.** (Support OoHPaG ed. Ottawa, Ontario, Canada: Royal College of Physicians and Surgeons of Canada; 2009.

345. Royal College of Physicians and Surgeons of Canada (RCPSC): **Cutting Through the Healthsystem Informaiton Fog: Royal College Environmental Scan.** 2012 edition. Ontario, Canada; 2012. http://www.royalcollege.ca/portal/page/portal/rc/common/documents/advocacy/enviroscan_e.pdf

346. *Runnels V, Labonte R, Packer C: **Reflections on the ethics of recruiting foreign-trained human resources for health.** *Human Resources for Health [Electronic Resource]* 2011, **9**. . http://human-resources-health.biomedcentral.com/articles/10.1186/1478-4491-9-2

347. Salma J: **Career Advancement and Education Opportunities: Experiences and Perceptions of Internationally-Educated Nurses.** University of Alberta, Nursing; 2009.

Additonal Table 1
Sources Retained for Data Extraction and Charting
*** Cited in Review**

348. Salma J, Hegadoren, K., Ogilvie, L.: **Career advancement and educational opportunities: experiences and perceptions of internationally educated nurses.** *Nursing Leadership* 2012, **25:**56-67.

349. Santa Mina EE, Eifert, C., Ireland, M., Fine, C., Wilson, G., Micevski, V., Wojtiuk, R., Valderrama, M.: **The development of an online instrument for prior learning assessment and recognition of internationally educated nurses: A pilot study.** *The International Review of Research in Open and Distance Learning* 2011, **12:**Paper 5.

350. Saskatchewan Ministry of Health: **Working Together: Saskatchewan's Health Workforce Action Plan Summary of Goals & Proposed Actions.** Regina, Saskatchewan, Canada: Saskatchewan Health; 2005. http://www.gov.sk.ca/news-archive/2005/12/14-1138-attachment.pdf

351. Saskatchewan Ministry of Health: **Physician Recruitment Strategy.** Saskatoon, Saskatchewan: Saskatchewan Ministry of Health; 2010. http://www.gov.sk.ca/adx/aspx/adxGetMedia.aspx?mediaId=810&PN=Shared

352. *Saskatchewan Registered Nurses Association [SRNA]: **Saskatchewan International Recruitment, Lessons Learned and Recommendations for the Future.** Saskatoon, Saskatchewan; 2008.

353. Saskatchewan Registered Nurses Association [SRNA]: **Final Project Evaluation Report-Internationally Educated Nurse Project.** SRNA; 2008.

354. Saunders P: **Internationally Educated Health Professionals in Nova Scotia: Why They Come, Why They Stay and the Challenges They Face.** Halifax, Nova Scotia; 2008. http://www.atlanticcanadahealthcare.com/images/pdf/Stories__Vocies_NS_08.pdf

355. Scarrow J: **Nursing in a new land.** *Registered Nurse Journal* 2008, **20:**22-23.

Additonal Table 1
Sources Retained for Data Extraction and Charting
*** Cited in Review**

356. Schwartz B: **Admitted but Excluded: Removing Occupatonal Barriers to Entry for Immigrants to Canada.** Winnipeg, Manitoba, Canada; 2012. https://ablb.mb.catalogue.libraries.coop/eg/opac/record/111005416

357. Sharieff W, Zakus, D. : **Resource utilization and costs borne by international medical graduates in their pursuit for practice license in Ontario, Canada.** *Pakistan Journal of Medical Sciences* 2006, **22:**109-115.

358. Shuchman M: **Searching for docs on foreign shores.** *Canadian Medical Association Journal* 2008, **178:**379-380.

359. Singh MD, Sochan, A.: **Voices of internationally educated nurses: policy recommendations for credentialing.** *International Nursing Review* 2010, **57:**56-63.

360. Slade S: **Ethical Recruitment of International Medical Graduates: Taking the Dialogue Forward.** Ottawa, Ontario, Canada; 2008. https://www.afmc.ca/pdf/DataPoint_Sept_2008_eng.pdf

361. *Sochan A, Singh,M. D.: **Acculturation and socialization: Voices of internationally educated nurses in Ontario.** *International Nursing Review* 2007, **54:**130-136.

362. Society of Rural Physicians of Canada: **International Medical Graduate Policy: Recommended Strategies.** Shawville, Quebec, Canada; 2002. https://www.srpc.ca/resources_library_enter.html

363. Sockalingam S, Hawa, R., Al-Battran, M., Abbey, S.E., Zaretsky, A.: **Preparing international medical graduates for psychiatry residency: A multi-site needs assessment.** *Academic Psychiatry* 2012, **36:**277-281.

364. Spurgeon D: **Canada waives examination rules for foreign cancer specialists.** *British Medical Journal: BMJ* 2000, **321:**1243.

Additonal Table 1
Sources Retained for Data Extraction and Charting
*** Cited in Review**

365. Standing Committee on Human Resources Skills and Social Development and the Status of Persons with Disabilities: **A Framework for Success: Practical Recommendations to Further Shorten the Foreign Qualification Recognition Process.** Ottawa, Ontario: House of Commmons of Canada; 2012.

366. Standing Senate Committee on Social Affairs SaT: **Time for Transformative Change. A Review of the @004 Health Accord.** Ottawa, Ontario; 2012. http://www.parl.gc.ca/content/sen/committee/411/soci/rep/rep07mar12-e.pdf

367. Stenerson HJ, Davis, P.M., Labash, A.M.: **Orientation of international medical graduates to Canadian medical practice.** *The Journal of Continuing Higher Education* 2012, **57:**29-34.

368. Szafran O, Crutcher, R.A., Banner, S.R. Watanabe, M: **Canadian and immigrant international medical graduates.** *Canadian Family Physician* 2005, **51:**1242.

369. Taylor A, Foster, J., Cambre, C.: **Training expendable workers: Temporary foreign workers in nursing.** *Globalisation, Societies and Education* 2012, **10:**95-117.

370. The Maytree Foundation: **Doctor Shortages and the Integration of International Physicians: Opportunties and Solutions.** Toronto, Ontario; 2001. . http://maytree.com/PDF_Files/SummaryDoctorShortagesAndTheIntegrationOfInternationalPhysicians2001.pdf

371. The University of British Columbia: **2012 Admissions Statistical Summary: International Dental Degree Completion Program** [Retrieved from: http://www.dentistry.ubc.ca/iddcp/iddcp-about/]

Additonal Table 1
Sources Retained for Data Extraction and Charting
*** Cited in Review**

372. Thind A, Freeman, T., Cohen, I., Thorpe, C., Burt, A., Stewart, M.: **Caractéristiques et pratiques médicales des diplômés en médecine de l'étranger. iffèrent-elles de celles des médecins formés au Canada?** *Canadian Family Physician* 2007, **53:**1330-1331.

373. Thind A, Feightner, J., Stewart, A., Thorpe, C., Burt, A.: **Who delivers preventive care as recommended? Analysis of physician and practice characteristics.** *Canadian Family Physician* 2008, **54:**1574-1575.

374. Tilley CM: **Support for internationally educated nurses transitioning into practice: An integrative literature review.** University of Victoria, Nursing; 2007.

375. Toguri C, Jong, M., Roger, J.: **Needs of specialists in rural and remote Canada.** *Canadian Journal of Rural Medicine* 2012, **17:**56-62.

376. Tomson G, Cohl, K.: **IMG Selection: An Independent Reivew of Access to Postgraduate Programs by International Medical Graduates in Ontario. Volume 1: Findings and Recommendaitons & Volume 2: Analysis and Background.** Toronto, Ontario, Canada; 2011.

377. *Tregunno D, Campbell H, Allen D, de Sousa D: **Internationally Educated Nurses (IEN) Knowledge Translation Project Report, Learn From My Experience.** Toronto, Ontario; 2007.

378. Tregunno D, Peters S, Campbell H, Gordon S: **International nurse migration: U-turn for safe workplace transition.** *Nursing Inquiry* 2009, **16:**182-190.

379. Truscott A: **Moratorium urged for foreign visa trainees.** *CMAJ: Canadian Medical Association Journal* 2008, **179:**638-639.

Additonal Table 1
Sources Retained for Data Extraction and Charting
*** Cited in Review**

380. Turner K: **Road to licensure for international educated nurses (IEN).** *SRNA Newsbulletin* 2009, **11:**22.

381. *Turrittin J, Hagey, R., Guruge, S., Collins, E., Mitchell, M.,: **The experiences of professional nurses who have migrated to Canada: Cosmopolitan citizenship of democratic racism?** *International Journal of Nursing Studies* 2002, **39**.

382. Urowitz MB: **Physician Assistant Opportunities for International Medical Graduates.** Toronto, Onatrio; 2008.

383. Vallevand A, Violato, C.: **A predictive and construct validity study of a high-stakes objective clinical examination for assessing the clinical competence of international medical graduates.** *Teaching and Learning in Medicine: An International Journal* 2012, **24:**169-176.

384. *Van Iterson L: **Blast off! The launch of** http://www.GoCanadaOT.com**.** *Occupational Therapy Now* 2010, **12:**35-25.

385. Van Iterson L: **The occupational therapy examination and practice preparation project (OTepp): Effective, popular and growing.** *Occupational Therapy Now* 2011, **13:**16-18.

386. Vandersloot: **The Lived-Experience of Internationally-trained midwives working as registered midwives in Ontario. .** University of Toronto Department of Adult Education and Counselling Psychology 2009.

387. Vardy D, Ryan, A., Audas, R.,: **Provisionaly licensed international medical gradutes: recruitment and retention in Newfoundland and Labrador.** *Our Diverse Cities* 2008, **5:**109-114.

388. von Zweck C: **Enabling the Workforce Integration of International Graduates: Issues and Recommendations for Occupational Therapy in Canada.** Ottawa, Ontario: Canadian Association of Occupational Therapists; 2006. https://www.caot.ca/pdfs/wip/WIP%20Report.pdf

Additonal Table 1
Sources Retained for Data Extraction and Charting
*** Cited in Review**

389. Waddell JP: **Physician Shortage.** *Canadian Journal of Surgery* 2006, **49:**236.

390. Walsh A, Banner S, Schabort I, Armson H, Bowmer MI, Granata B: **International Medical Graduates - Current Issues. .** The Association of Faculties of Medicine of Canada; The College of Family Physicians of Canada; Le Collège des médecins du Québec; and, The Royal College of Physicians and Surgeons of Canada. All Rights Reserved. 2011. https://www.afmc.ca/pdf/fmec/05_Walsh_IMG%20Current%20Issues.pdf

391. Ward T: **Is There Light at the End of the Tunnel-Can We Resolve the Physician Distribution Challenge in Canada?** (CEO Pa ed. Scarborough, Ontario, Canada: The Scarborough Hospital; 2009.

392. Watanabe M: **Analysis of international migration patterns affecting physician supply in Canada.** *Nurisng Leadership* 2008, **3:**e129-e138.

393. Watt D, Violato, C., Lake, D., Baig, L.: **Effectiveness of a clinically relevant education program for improving medical communication and clinical skills of international medical graduates.** *2010* 2010, **1:**e70-e80. http://communication4integration.ca/wp-content/uploads/2013/05/MCAPFinalReport2010_03Oct2011.pdf

394. Watt D, Violato, C., Lake, D: **A Longitudinal cross-Sequential Study of the Professional Integration of International Medical Graduates (IMGs) from Application to Licensure.** In *Final Report*. Calgary, Alberta, Canada: Univresity of Calgary; 2012.

395. Watt DLE, Lake, D., Cabrnoch, T., Leonard, K.: **Assessing English Language Proficiency of International Medical Graduates in their Integration into Canada's Physician Supply.** Ottawa, Canada; 2003.

Additonal Table 1
Sources Retained for Data Extraction and Charting
*** Cited in Review**

396. Watt DLE, Violato, C., Lake, D.M.: **Medical Communication Assessment Project. Final Report 2010.** Calgary, Alberta, Canada; 2010.

397. Watts E, Davies, J.C., Metcalfe, D.: **The Canadian International Medical Graduate Bottleneck: A New Problem for New Doctors.** *Canadian Medical Education Journal* 2011, **2:**e86-e90.

398. Weerasekera P: **Psychotherapy training for IMGs: Attending to the "How to" and "What to" teach.** *Academic Psychiatry* 2012, **36:**288-292.

399. Western Alliance for Assessment of International Physicians [WAAIP]: **WAAIP Final Progress Report for October 1, 2005 - May 31, 2006.** 2006. www.hhrpforum.com

400. Western and Northern Health Human Resources and Planning Forum: **Literature Review of Issues Relating to the Needs, Challenges and Successes Relating to Integration of Internationally Educated Health Professionals (IEHP).** Victoria, British Columbia; 2006. www.hhrpforum.com

401. Western and Northern Health Human Resources Planning Forum: **An Environmental Scan of Programs and Services available to Internationally Educated Healthcare Professionals in Canda.** Victoria, British Columbia; 2006. www.hhrpforum.com

402. Wharry S: **Pressue mounting to curb MD poaching by rich nations.** *CMAJ: Canadian Medical Assocatin Journal* 2002, **166:**1701.

403. Wong A, Lohfeld, L.: **Recertifying as a doctor in Canada: international medical graduates and the journey from entry to adaptation.** *Medical Education* 2008, **Education:**1.

Additonal Table 1
Sources Retained for Data Extraction and Charting
*** Cited in Review**

404. Xu Y, Zhang, J.: **One size doesn't fit all: Ethics of international nursing recruitment from the conceptual framework of stakeholder interests.** *Nursing Ethics* 2005, **12:**571-581.

405. Yan J: **Health services delivery: reframing policies for global nursing migration in North America--a Caribbean perspective.** *Policy, Politics & Nursing Practice* 2006, **7:**71S-75S.

406. *Zaman H: *Breaking the Iron Wall: De-Commodificaiton and Immigrant Women's labour in Canada.* Lanham, Maryland: Lexington Books; 2006.

407. Zulla R, Baerlocher, M.O., Verma, S.: **International medical graduates (IMGs) needs assessment study: comparison between current IMG trainees and program directors.** *BMC Medical Education* 2008, **8**.
