# Supplementary material for: Scoping review about the professional integration of internationally educated health professionals
Source: Hum Resour Health. 2016 Jun 17;14:38. doi: 10.1186/s12960-016-0135-6 (PMC4912807; doi:10.1186/s12960-016-0135-6)
Supplement: Additional file 2: — Thematic coverage per source. (PDF 596 kb) [file 12960_2016_135_MOESM2_ESM.pdf]

Additional Table 2.
Thematic Coverage per Source

	Source First Author (Year)	Location	Type of Paper	Methods	Profession	Themes				
						Pre-Immigration Activities & Programs	Early Arrival Activities & Programs	Professional Recertification	Alternative Paths to Integration	Workplace Integration
1.	Advisory Committee on Health Delivery and Human Resources [ACHDHR] (2010)	Pan Canadian	Empirical	Mixed Methods	IEHPs	✓				
2.	Agbassi & Don-Wauchope (2010)	Ontario	Empirical	Qualitative	IEMLT					✓
3.	Alberta International Medical Graduates Association [AIMGA] (2012)	Alberta	Non Empirical		IMGs					✓
4.	Alberta Network of Immigrant Women [ANIW (2002)	Alberta	Empirical	Mixed Methods	IENs	✓				
5.	Alberta Network of Immigrant Women [ANIW] (2005)	Alberta	Empirical	Qualitative	IENs			✓		
6.	Andrew (2010)	Ontario	Empirical	Quantitative	IMGs			✓		
7.	Association of Canadian Community Colleges (2012)	Pan Canadian	Non Empirical		IEHPs			✓		
8.	Association of Faculties of Medicine of Canada (2011)	Pan Canadian	Empirical	Quantitative	IMGs			✓		
9.	Association of International Physicians & Surgeons of Ontario [AIPSO] (2000)	Ontario	Non Empirical		IMGs		✓			
10.	Association of International Physicians & Surgeons of Ontario [AIPSO] (2002)	Ontario	Non Empirical		IMGs			✓		
11.	Association of International Physicians and Surgeons of Ontario [AIPSO] (2012)	Ontario	Non Empirical		IMGS			✓		
12.	Atack et al. (2012)	Ontario	Non Empirical		IENs			✓		
13.	Atlin (2002)	Pan Canadian	Non Empirical		IMGs		✓			

Additional Table 2.
Thematic Coverage per Source

Source First Author (Year)		Location	Type of Paper	Methods	Profession	Themes				
						Pre-Immigration Activities & Programs	Early Arrival Activities & Programs	Professional Recertification	Alternative Paths to Integration	Workplace Integration
14.	Audas, Ross & Vardy (2004)	Newfoundland & Labrador	Non Empirical		IMGs					✓
15.	Audas et al. (2005)	Pan Canadian	Non Empirical		IMGs		✓			
16.	Audas et al. (2009)	Newfoundland & Labrador	Empirical	Quantitative	IMGs		✓			
17.	Austin et al. (2003)	Ontario	Empirical	Quantitative	ITPs		✓			
18.	Austin (2003)	Ontario	Empirical	Mixed Methods	ITPs					✓
19.	Austin (2005)	Ontario	Empirical	Qualitative	ITPs					✓
20.	Austin & Rocchi Dean (2006)	Ontario	Non Empirical		ITPs			✓		✓
21.	Austin & Croteau (2007)	Ontario	Non Empirical		ITPs			✓		✓
22.	Austin, Martin & Gregory (2007)	Ontario	Empirical	Quantitative	ITPs				✓	
23.	Austin (2007)	Ontario	Empirical	Quantitative	ITPs				✓	
24.	Austin, Gregory & Galli (2008)	Pan Canadian	Empirical	Quantitative	ITPs				✓	
25.	Austin (2008)	Ontario	Non Empirical		IEHPs				✓	
26.	Austin & Ensom (2008)	Pan Canadian	Non Empirical		ITPs					✓
27.	Baerlocher (2006)	Pan Canadian	Empirical	Quantitative	IMGs					✓

Additional Table 2.
Thematic Coverage per Source

Source First Author (Year)		Location	Type of Paper	Methods	Profession	Themes				
						Pre-Immigration Activities & Programs	Early Arrival Activities & Programs	Professional Recertification	Alternative Paths to Integration	Workplace Integration
28.	Baig, Violato & Crutcher (2009)	Pan Canadian	Empirical	Quantitative	IMGs			✓		
29.	Baldacchino & Hood (2008a)	Pan Canadian	Empirical	Qualitative	IEHPs			✓		
30.	Baldacchino & Hood (2008b)	Prince Edward Island	Empirical	Mixed Methods	IEHPs			✓		✓
31.	Baldacchino & Saunders (2010)	Atlantic Provinces	Empirical	Qualitative	IEHPs					✓
32.	Banerjee (2010)	Saskatchewan	Non Empirical		IENs			✓		
33.	Banner, Bowmer & Rattanasithy (2011)	Pan Canadian	Empirical	Quantitative	IMGs			✓		
34.	Baptiste et al. (2010)	Ontario	Non Empirical		IEHPs (IEPT & IEOT)			✓		
35.	Bard (2009)	Pan Canadian	Non Empirical		IENs			✓		
36.	Baringhausen & Bloom (2009)	Pan Canadian	Non Empirical		IEHPs	✓				
37.	Barry et al. (2003)	Pan Canadian	Non Empirical		IENs		✓			
38.	Bassendowski & Petrucka (2010)	Ontario	Empirical	Quantitative	IENs	✓				
39.	Bates & Andrew (2001)	Pan Canadian	Non Empirical		IMGs			✓		
40.	Baumann, Blythe & Kolotylo (2004a)	Pan Canadian	Empirical	Mixed Methods	IENs	✓				
41.	Baumann et al. (2004)	Pan Canadian	Non Empirical		IENs	✓		✓		✓

Additional Table 2.
Thematic Coverage per Source

	Source First Author (Year)	Location	Type of Paper	Methods	Profession	Themes: Pre-Immigration Activities & Programs	Early Arrival Activities & Programs	Professional Recertification	Alternative Paths to Integration	Workplace Integration
42.	Baumann et al. (2006)	Ontario	Non Empirical		IENs	✓				
43.	Baumann, Blythe & Kolotylo (2006)	Pan Canadian	Non Empirical		IENs	✓				✓
44.	Baumann & Blythe (2009a)	Ontario	Empirical	Mixed Methods	IENs	✓	✓			
45.	Bauman, Blythe & Ross (2010)	Ontario	Non Empirical		IEHPs		✓			
46.	Baumann, Blythe & Hunsberger (2010)	Ontario	Non Empirical		IEHPS			✓		✓
47.	Baumann & Blythe (2012a)	Ontario	Non Empirical		IENs		✓			
48.	Baumann & Blythe (2012b)	Ontario	Non Empirical		IENs			✓		✓
49.	BCMA Council on Health Economics and Policy (2011)	British Columbia	Empirical	Quantitative	IMGs			✓		
50.	Beaton & Walsh (2010)	Newfoundland & Labrador	Empirical	Qualitative-Oral History	IENs	✓	✓	✓		✓
51.	Beck et al. (2008)	Pan Canadian	Non Empirical		IEMs			✓		
52.	Belkhodja et al. (2009)	Pan Canadian	Empirical	Mixed Methods	IEHPs			✓		
53.	Beran et al. (2012)	Alberta	Empirical	Quantitative	IMGs			✓		
54.	Bhimji (2010)	Alberta	Non Empirical		IMGs			✓		
55.	Blais (2008)	Pan Canadian	Empirical	Mixed Methods	ITMRTs			✓		

Additional Table 2.
Thematic Coverage per Source

Source First Author (Year)		Location	Type of Paper	Methods	Profession	Themes: Pre-Immigration Activities & Programs	Early Arrival Activities & Programs	Professional Recertification	Alternative Paths to Integration	Workplace Integration
56.	Blais & Darling (2009)	Pan Canadian	Non Empirical		ITMRTs			✓		
57.	Blooberg , Schonwetter & Swain (2009)	Pan Canadian	Empirical	Mixed Methods	ITDs				✓	
58.	Blythe & Baumann (2008)	Ontario	Non Empirical		IENs					✓
59.	Blythe & Baumann (2009)	Ontario	Empirical	Quantitative	IENs					✓
60.	Blythe et al. (2009)	Ontario	Empirical	Qualitative	IENs					✓
61.	Bobrosky (2010)	Pan Canadian	Review		IMGS				✓	
62.	Boschma & Santiago (2012)	Pan Canadian	Non Empirical		IENs					✓
63.	Bourgeault et al. (2010)	Pan Canadian	Empirical	Qualitative	IEHPs		✓	✓	✓	
64.	Bourgeault (2006)	Pan Canadian	Empirical	Qualitative	IEHPs (IEMs, IENs, IMGs)	✓	✓	✓	✓	✓
65.	Bourgeault (2007)	Pan Canadian	Non Empirical		IEHPs	✓		✓		
66.	Bourgeault et al. (2009)	Pan Canadian	Empirical	Mixed Methods	IEHPs	✓	✓	✓		✓
67.	Bourgeault, Neiterman & LeBrun (2010)	Pan Canadian	Empirical	Qualitative	IEMs			✓		
68.	Bourgeault et al. (2011)	Pan Canadian	Non Empirical		IEHPs			✓		
69.	Bourgeault & Baumann (2011)	Pan Canadian	Empirical	Qualitative	IEHPs	✓				

Additional Table 2.
Thematic Coverage per Source

Source<br>First Author (Year)		Location	Type of Paper	Methods	Profession	Themes				
						Pre-Immigration Activities & Programs	Early Arrival Activities & Programs	Professional Recertification	Alternative Paths to Integration	Workplace Integration
70.	Bourgeault (2012)	Pan Canadian	Non Empirical		IENs	✓				
71.	Bowmer (2009)	Pan Canadian	Empirical	Mixed Methods	IEHPs				✓	
72.	Boyd & Schellenberg (2008)	Pan Canadian	Empirical	Quantitative	IMGs					✓
73.	British Columbia Health Professions Review Board Stakeholder Consultation Committee (2010)	British Columbia	Empirical	Qualitative	IEHPs			✓		
74.	Broten (2008)	Pan Canadian	Non Empirical		IMGs			✓		
75.	Brown & Raborn (2001)	Pan Canadian	Empirical	Quantitative	IMGs			✓		
76.	Brownell (2011)	Pan Canadian	Empirical	Qualitative	IMGs			✓		
77.	Bruce & Zwicker (2008)	Atlantic Provinces	Empirical	Mixed Methods	IEHPs					✓
78.	Brynaert & Associates (2010)	Ontario	Empirical	Qualitative	IEHPs			✓	✓	
79.	Buchan (2001)	Pan Canadian	Non Empirical		IENs	✓				
80.	Buhr (2006)	Ontario	Empirical	Quantitative	IENs			✓		✓
81.	Buske (2000)	Pan Canadian	Non Empirical		IMGs			✓		
82.	Buske & Slade (2009)	Pan Canadian	Empirical	Quantitative	IMGs					✓
83.	Buske (2010)	Pan Canadian	Empirical	Qualitative	IMGs			✓		
84.	Cadieux et al. (2007)	Quebec	Empirical	Quantitative	IMGs					✓

Additional Table 2.
Thematic Coverage per Source

Source First Author (Year)		Location	Type of Paper	Methods	Profession	Themes				
						Pre-Immigration Activities & Programs	Early Arrival Activities & Programs	Professional Recertification	Alternative Paths to Integration	Workplace Integration
85.	Cameron, Este & Worthington (2000)	Alberta	Empirical	Qualitative	IEHPs (IENs, IMGs, ITPs)			✓		✓
86.	Canadian Alliance of Physiotherapy Regulators & Canadian Physiotherapy Association (2002)	Pan Canadian	Empirical	Qualitative	IEPTs			✓		
87.	Canadian Association of Medical Radiation Technologist (2012)	Pan Canadian	Non Empirical		IEMRTs			✓		
88.	Canadian Association of Medical Radiation Technologist (2006)	Pan Canadian	Non Empirical		IEMRTs			✓		
89.	Canadian Association of Occupational Therapists (2007)	Pan Canadian	Non Empirical		IEOTs		✓	✓		
90.	Canadian Association of Occupational Therapists (2012)	Pan Canadian	Non Empirical		IEOTs			✓		
91.	Canadian Association of Physiotherapist Regulators (2012)	Pan Canadian	Non Empirical		IEPTs			✓		
92.	Canadian Association of Schools of Nursing (CASN) (2012)	Pan Canadian	Non Empirical		IENs			✓		
93.	Canadian Collaborative Centre for Physician Resources (2008)	Pan Canadian	Empirical	Quantitative	IMGs			✓		
94.	Canadian Federation of Nurses Unions (2009)	Pan Canadian	Non Empirical		IENs	✓				
95.	Canadian Institute for Health Information (2011)	Pan Canadian	Empirical	Quantitative	IENs					✓

Additional Table 2.
Thematic Coverage per Source

Source<br>First Author (Year)		Location	Type of Paper	Methods	Profession	Themes				
						Pre-Immigration Activities & Programs	Early Arrival Activities & Programs	Professional Recertification	Alternative Paths to Integration	Workplace Integration
96.	Canadian Institute for Health Information (2012)	Pan Canadian	Empirical	Quantitative	IENs					✓
97.	Canadian Institute for Health Information: Summary (2007)	Pan Canadian	Empirical	Mixed Methods	IEHPs					✓
98.	Canadian Institute for Health Information: Workforce (2007)	Pan Canadian	Empirical	Quantitative	IENs					✓
99.	Canadian Institute for Health Information: Internationally (2007)	Pan Canadian	Non Empirical		IEHPs (IENs, IMGs)					✓
100.	Canadian Institute for Health Information: Canada's (2008)	Pan Canadian	Empirical	Mixed Methods	IEHPs					✓
101.	Canadian Institute for Health Information: Regulated (2008)	Pan Canadian	Empirical	Quantitative	IMGs					✓
102.	Canadian Institute for Health Information (2009)	Pan Canadian	Empirical	Quantitative	IENs					✓
103.	Canadian Institute for Health Information (2010)	Pan Canadian	Empirical	Quantitative	IENs					✓
104.	Canadian Institute for Health Information Canada's (2007)	Pan Canadian	Empirical	Quantitative	IEHPs					✓
105.	Canadian Institute for Health Information (2007)	Pan Canadian	Non Empirical		IMGs					✓
106.	Canadian Institute for Health Information (2009)	Pan Canadian	Non Empirical		IMGs					✓
107.	Canadian Institute for Health Information (2012a)	Pan Canadian	Non Empirical		IEOTs					✓
108.	Canadian Institute for Health Information (2012b)	Pan Canadian	Empirical	Quantitative	IMGs					✓

Additional Table 2.
Thematic Coverage per Source

	Source First Author (Year)	Location	Type of Paper	Methods	Profession	Themes: Pre-Immigration Activities & Programs	Early Arrival Activities & Programs	Professional Recertification	Alternative Paths to Integration	Workplace Integration
109.	Canadian Labour and Business Centre (2003)	Pan Canadian	Non Empirical		IMGs					✓
110.	Canadian Medical Association (2004)	Pan Canadian	Empirical	Mixed Methods	IMGs			✓		
111.	Canadian Medical Laboratory Technologist Society (2009)	Pan Canadian	Non Empirical		IEMLTs			✓		
112.	Canadian Nurses Association (2009a)	Pan Canadian	Non Empirical		IENs			✓		
113.	Canadian Nurses Association (2009b)	Pan Canadian	Non Empirical		IENs	✓				
114.	Canadian Nurses Association & Canadian Medical Association (2005)	Pan Canadian	Non Empirical		IEHPs (IENs, IMGs)			✓		
115.	Canadian Post-M.D. Education Registry (2006)	Pan Canadian	Non Empirical		IMGs		✓	✓		✓
116.	Canadian Society of Medical Laboratory Sciences (2011)	Pan Canadian	Non Empirical		IEMLTs			✓		
117.	Canadian Society of Medical Laboratory Sciences (2009)	Pan Canadian	Non Empirical		IEMLTs			✓		
118.	Carter, Morrish & Amoyaw (2008)	Manitoba	Non Empirical		IEHPs		✓			
119.	Carter et al. (2009)	Manitoba	Empirical	Mixed Methods	IEHPs	✓	✓			
120.	Cartmel (2009)	British Columbia	Non Empirical		IENs			✓		
121.	Cayabyab (2010)	Saskatchewan	Non Empirical		IENs			✓		

Additional Table 2.
Thematic Coverage per Source

	Source First Author (Year)	Location	Type of Paper	Methods	Profession	Themes: Pre-Immigration Activities & Programs	Early Arrival Activities & Programs	Professional Recertification	Alternative Paths to Integration	Workplace Integration
122.	Centre for Internationally Educated Nurses (CARE) (2012)	Ontario	Non Empirical		IENs			✓		
123.	Centre for the Evaluation of Health Professionals Educated Abroad (2012)	Ontario	Empirical	Quantitative	IMGs			✓		✓
124.	Chan (2002)	Pan Canadian	Empirical	Quantitative	IMGs					✓
125.	Chew et al. (2010)	British Columbia	Non Empirical		IMGs			✓		
126.	Childs & Herbert (2007)	Ontario	Empirical	Quantitative	IMGs			✓		
127.	Cho et al. (2011)	Pan Canadian	Empirical	Quantitative	IENs					✓
128.	Choi (2005)	Pan Canadian	Non Empirical		IENs			✓		
129.	Cholakis (2005)	Pan Canadian	Non Empirical		ITDs					✓
130.	Citizenship and Immigration Canada (2011)	Pan Canadian	Non Empirical		IEHPs		✓			
131.	Citizenship and Immigration Canada (2012)	Pan Canadian	Non Empirical		IEHPS	✓				
132.	Coffey (2006)	Ontario	Non Empirical		IENs				✓	
133.	College of Family Physicians of Canada (2004)	Ontario	Non Empirical		IMGs			✓		
134.	College of Physicians and Surgeons of Ontario (2004)	Ontario	Non Empirical		IMGs			✓		
135.	College of Physicians and Surgeons of Ontario (2007)	Ontario	Non Empirical		IMGs					✓

Additional Table 2.
Thematic Coverage per Source

	Source<br>First Author (Year)	Location	Type of Paper	Methods	Profession	Themes				
						Pre-Immigration Activities & Programs	Early Arrival Activities & Programs	Professional Recertification	Alternative Paths to Integration	Workplace Integration
136.	College of Physicians and Surgeons of Ontario (2008)	Ontario	Non Empirical		IMGs			✓		
137.	College of Registered Nurses of Nova Scotia (2010a)	Nova Scotia	Non Empirical		IENs			✓		
138.	College of Registered Nurses of Nova Scotia (2010b)	Nova Scotia	Non Empirical		IENs			✓		
139.	Collins (2004)	Ontario	Empirical	Qualitative	IENs			✓		✓
140.	Comeau (2009)	Pan Canadian	Empirical	Quantitative	IMGs			✓		✓
141.	Consortium National de Formation en Sante (2008a)	Pan Canadian	Non Empirical		IEHPs			✓		
142.	Consortium National de Formation en Sante (2008b)	Pan Canadian	Non Empirical		IEHPs			✓		
143.	Craig, Brick & Carlik (2002)	Pan Canadian	Empirical	Quantitative	IMGs			✓		
144.	Crutcher et al. (2003)	Pan Canadian	Empirical	Quantitative	IMGs			✓		
145.	Crutcher et al. (2007)	Pan Canadian	Non Empirical		IMGs			✓		
146.	Crutcher et al. (2011)	Alberta	Empirical	Quantitative	IMGs		✓			
147.	Curran et al. (2008a)	Newfoundland & Labrador	Empirical	Qualitative	IMGs					✓
148.	Curran et al. (2008b)	Newfoundland & Labrador	Empirical	Quantitative	IMGs			✓		
149.	Cutcliffe et al. (2011)	Pan Canadian	Non Empirical		IENs	✓				
150.	Dauphinee (2005)	Pan Canadian	Non Empirical		IMGs					✓

Additional Table 2.
Thematic Coverage per Source

Source First Author (Year)		Location	Type of Paper	Methods	Profession	Themes				
						Pre-Immigration Activities & Programs	Early Arrival Activities & Programs	Professional Recertification	Alternative Paths to Integration	Workplace Integration
151.	Dauphinee (2006)	Pan Canadian	Empirical	Quantitative	IMGs					✓
152.	Dauphinee & Buske (2006)	Pan Canadian	Empirical	Qualitative	IMGs					✓
153.	Davis (2002)	Pan Canadian	Non Empirical		MLT		✓			
154.	de Carvalho (2007)	Pan Canadian	Empirical	Qualitative	IMGs	✓				
155.	Deber (2010)	Pan Canadian	Non Empirical		IEHPs	✓				
156.	Dhalla & Born (2011)	Pan Canadian	Non Empirical		IMGs			✓		
157.	Dickson (2007)	Newfoundland & Labrador	Empirical	Quantitative	IMGs	✓	✓	✓		
158.	Dodani & LaPorte (2005)	Pan Canadian	Non Empirical		IEHPs	✓				
159.	Dore et al. (2010)	Pan Canadian	Empirical	Quantitative	IMGs			✓		
160.	Dove (2009)	Pan Canadian	Non Empirical		IMGs	✓		✓		✓
161.	Doyle (2010)	Pan Canadian	Non Empirical		IMGs			✓		
162.	Driscoll (2009)	Pan Canadian	Non Empirical		IMGs			✓		
163.	Duff (2002)	Ontario	Empirical	Quantitative	IEHPs					✓
164.	Dumont, Zurn & Church (2008)	Pan Canadian	Non Empirical		IEHPs (IENs, IMGs)			✓	✓	✓
165.	Duncan, Poisson & Wong (2008)	Pan Canadian	Empirical	Qualitative	IENs			✓		

Additional Table 2.
Thematic Coverage per Source

	Source First Author (Year)	Location	Type of Paper	Methods	Profession	Themes: Pre-Immigration Activities & Programs	Early Arrival Activities & Programs	Professional Recertification	Alternative Paths to Integration	Workplace Integration
166.	Dywili et al. (2012)	Pan Canadian	Non Empirical		IEHPs (IEHPs & IMGs)					✓
167.	Elgersma (2012)	Pan Canadian	Non Empirical		IEHPs			✓		
168.	Emery & Ferrer (2010)	Alberta	Non Empirical		IEHPs		✓			
169.	Federal/Provincial/Territorial Advisory Committee on Health Delivery and Human Resources (2009)	Pan Canadian	Non Empirical		IEHPs	✓				
170.	Fleming & Mathews (2012)	Newfoundland & Labrador	Empirical	Quantitative	IMGs					✓
171.	Fooks & Maslove (2004)	Pan Canadian	Non Empirical		IEHPs (IENs, IMGs, ITPs)	✓				
172.	Foster (2008)	Pan Canadian	Non Empirical		IMGs			✓		
173.	Frank & Saunders (2008)	Nova Scotia	Empirical	Qualitative	IMGs	✓				
174.	Gallant (2011)	Newfoundland & Labrador	Empirical	Mixed methods	IENs					✓
175.	Garibaldi et al. (2002)	Pan Canadian	Empirical	Quantitative	IMGs			✓		
176.	Glover Takahashi et al. (2008)	Ontario	Non Empirical		IEOTs			✓		
177.	Glover Takahashi, McIlory & Begs (2011)	Pan Canadian	Non Empirical		IEOTs			✓		
178.	Goldszmidt, Kortas & Meehan (2007)	Pan Canadian	Empirical	Quantitative	IMGs			✓	✓	

Additional Table 2.
Thematic Coverage per Source

	Source First Author (Year)	Location	Type of Paper	Methods	Profession	Themes: Pre-Immigration Activities & Programs	Themes: Early Arrival Activities & Programs	Themes: Professional Recertification	Themes: Alternative Paths to Integration	Themes: Workplace Integration
179.	Government of Alberta (2006)	Alberta	Non Empirical		IEHPs (IEHPs & IMGs)	✓				
180.	Government of Nova Scotia (2005)	Nova Scotia	Non Empirical		IEHPs		✓			
181.	Government of Ontario (2005)	Ontario	Non Empirical		IEHPs		✓			
182.	Grant (2006)	Pan Canadian	Non Empirical		IMGs		✓			✓
183.	Grant (2009a)	Pan Canadian	Non Empirical		IEMLTs			✓		
184.	Grant (2010a)	Pan Canadian	Non Empirical		IEMLTs			✓		✓
185.	Grant (2008)	Pan Canadian	Non Empirical		IEMLTs			✓	✓	
186.	Grant et al. (2008)	Pan Canadian	Non Empirical		IEMLTs			✓		
187.	Grant (2009b)	Pan Canadian	Non Empirical		IEMLTs			✓	✓	
188.	Grant (2009c)	Pan Canadian	Non Empirical		IEMLTs			✓		
189.	Grant (2010b)	Pan Canadian	Empirical	Quantitative	MLT			✓		✓
190.	Grant (2010c)	Pan Canadian	Non Empirical		IEMLTs			✓		

Additional Table 2.
Thematic Coverage per Source

Source First Author (Year)		Location	Type of Paper	Methods	Profession	Themes				
						Pre-Immigration Activities & Programs	Early Arrival Activities & Programs	Professional Recertification	Alternative Paths to Integration	Workplace Integration
191.	Grant (2011)	Pan Canadian	Empirical	Quantitative	IEMLTs					✓
192.	Gregory (2011)	Saskatchewan	Empirical	Quantitative	IENs			✓		
193.	Griffiths (2001)	British Columbia	Non Empirical		IENs					✓
194.	Gushue (2000)	Newfoundland & Labrador	Non Empirical		IMGs			✓		
195.	Hagley et al. (2001)	Pan Canadian	Empirical	Qualitative	IENs					✓
196.	Haley & Simosko (2011)	Pan Canadian	Empirical	Mixed Methods	IEMLTs		✓	✓		
197.	Hall et al. (2004)	Ontario	Empirical	Qualitative	IMGs			✓		
198.	Hamilton (2008)	Saskatchewan	Non Empirical		IENs			✓		
199.	Hamilton (2009)	Alberta	Non Empirical		IENs			✓		
200.	Health Canada (2004)	Pan Canadian	Non Empirical		IMGs					✓
201.	Health Canada (2010)	Pan Canadian	Empirical	Mixed Methods	IEHPs			✓		
202.	HealthForceOntario (2009)	Ontario	Empirical	Quantitative	IMGs			✓		
203.	HealthForceOntario (2012)	Pan Canadian	Non Empirical		IEHPs		✓	✓		
204.	Hearnden (2007)	Ontario	Empirical	Mixed Methods	IENs					✓
205.	Hefley, Mandel & Gerace (2010)	Ontario	Non Empirical		IMGs			✓		

Additional Table 2.
Thematic Coverage per Source

	Source First Author (Year)	Location	Type of Paper	Methods	Profession	Themes				
						Pre-Immigration Activities & Programs	Early Arrival Activities & Programs	Professional Recertification	Alternative Paths to Integration	Workplace Integration
206.	Hewitt Associates (2010)	Newfoundland & Labrador	Non Empirical		IENs			✓		
207.	Higginbottom (2010)	Alberta	Empirical	Qualitative	IENs	✓				
208.	Higginbottom (2011)	Alberta	Empirical	Qualitative	IENs	✓		✓		
209.	Hoag (2008)	Pan Canadian	Non Empirical		IEHPs					✓
210.	Hofmeister, Lockyer & Crutcher (2009)	Alberta	Empirical	Quantitative	IMGs			✓		
211.	Human Resources and Skills Development Canada (2010)	Pan Canadian	Empirical	Mixed Methods	IEHPs			✓		
212.	Humber & Frecker (2008)	British Columbia	Empirical	Mixed Methods	IMGs	✓				
213.	Isreal (2011)	Ontario	Non Empirical		IEHPs (IED & IEHPs)			✓		
214.	Jablonski (2012)	Ontario	Empirical	Mixed Methods	IMGs		✓			
215.	Jacob et al. (2011)	Ontario	Empirical	Quantitative	IMGs					✓
216.	Jain et al. (2012)	Ontario	Non Empirical		IMGs			✓		
217.	Jeans, Hadley & Green (2005)	Pan Canadian	Empirical	Mixed Methods	IENs			✓		
218.	Jeans (2006)	Pan Canadian	Non Empirical		IENs	✓		✓		
219.	Johnson (2007)	Pan Canadian	Non Empirical		IEPTs			✓		

Additional Table 2.
Thematic Coverage per Source

	Source First Author (Year)	Location	Type of Paper	Methods	Profession	Themes				
						Pre-Immigration Activities & Programs	Early Arrival Activities & Programs	Professional Recertification	Alternative Paths to Integration	Workplace Integration
220.	Johnson (2011)	Pan Canadian	Non Empirical		IEPTs			✓		✓
221.	Johnson & Israel (2011)	Pan Canadian	Empirical	Mixed Methods	IERTs			✓		
222.	Johnston (2007)	Pan Canadian	Non Empirical		IMGs			✓		
223.	Joudrey (2010)	Pan Canadian	Empirical	Quantitative	IMGs	✓				
224.	Kabene, Howard & Zhou (2009)	Pan Canadian	Non Empirical		IMGs	✓				
225.	Katikireddi (2005)	Pan Canadian	Non Empirical		IMGs	✓				
226.	Kawi (2009)	Pan Canadian	Non Empirical		IENs					✓
227.	Keatings (2006)	Ontario	Non Empirical		IENs	✓		✓		
228.	Keenan (2005)	Pan Canadian	Non Empirical		IMGs			✓		
229.	Khaliq, Broyles & Mwachofi (2009)	Pan Canadian	Non Empirical		IENs	✓				
230.	Khan et al. (2006)	Pan Canadian	Empirical	Quantitative	IMGs					✓
231.	Klein et al. (2009)	Alberta	Empirical	Qualitative	IMGs	✓				
232.	Kline (2003)	Pan Canadian	Non Empirical		IENs	✓				
233.	Ko et al. (2005)	Pan Canadian	Empirical	Quantitative	IMGs					✓
234.	Kogo (2009)	Saskatchewan	Empirical	Mixed Methods	IMGS	✓				

Additional Table 2.
Thematic Coverage per Source

	Source First Author (Year)	Location	Type of Paper	Methods	Profession	Themes: Pre-Immigration Activities & Programs	Early Arrival Activities & Programs	Professional Recertification	Alternative Paths to Integration	Workplace Integration
235.	Kogon & Sandhu (2011)	Ontario	Empirical	Quantitative	IEHPs (IEHPs & ITD)			✓		
236.	Kolawole (2009)	Ontario	Non Empirical		IENs			✓		
237.	Kolawole (2010)	Ontario	Non Empirical		IENs			✓		
238.	Komarnicki (2012)	Pan Canadian	Non Empirical		IEHPs	✓	✓	✓		
239.	Kondro (2002)	Pan Canadian	Non Empirical		IMGs			✓		
240.	Kondro (2006a)	Pan Canadian	Non Empirical		IMGs			✓		
241.	Kondro (2006b)	Pan Canadian	Non Empirical		IMGs			✓		
242.	Kondro (2007)	Pan Canadian	Non Empirical		IMGs					✓
243.	Kondro (2009)	Pan Canadian	Empirical	Quantitative	IMGs					✓
244.	Kroeker (2008)	Manitoba	Non Empirical		IENs	✓	✓	✓		
245.	Labonte, Packer & Klassen (2006)	Pan Canadian	Empirical		IEHPs	✓				
246.	Lafontant et al. (2006)	Pan Canadian	Report Empirical	Mixed Methods	IEHPs			✓		
247.	Landry, Gupta & Tepper (2010)	Pan Canadian	Non Empirical		IEHPs					✓
248.	Lax et al. (2009)	Pan Canadian	Empirical	Quantitative	IMGs					✓
249.	Lindberg (2008)	Saskatchewan	Empirical	Qualitative	IENs					✓

Additional Table 2.
Thematic Coverage per Source

Source First Author (Year)		Location	Type of Paper	Methods	Profession	Themes				
						Pre-Immigration Activities & Programs	Early Arrival Activities & Programs	Professional Recertification	Alternative Paths to Integration	Workplace Integration
250.	Little (2007)	Pan Canadian	Non Empirical		IENs	✓				
251.	Lockyer et al. (2006)	Pan Canadian	Empirical	Quantitative	IMGs			✓		✓
252.	Lockyer et al. (2007)	Alberta	Empirical	Qualitative	IMGs			✓		✓
253.	Lockyer et al. (2010)	Pan Canadian	Empirical	Qualitative	IMGs			✓		✓
254.	Loewen et al. (2012)	Pan Canadian	Empirical	Quantitative	IMGs					✓
255.	Lombard (2005)	Nova Scotia	Empirical	Qualitative	IMGs					✓
256.	Lum (2009)	Ontario	Empirical	Qualitative	IEHPs (IENs, ITPs)				✓	
257.	Maamoun (2007)	Pan Canadian	Empirical	Mixed Methods	IERT					
258.	MacDonald-Rencz & Davis (2010)	Pan Canadian	Non Empirical		IEHPs			✓		✓
259.	MacLean (2010)	Pan Canadian	Non Empirical		IMGs	✓				
260.	MacLellan et al. (2012)	Quebec	Empirical	Quantitative	IMGs			✓		
261.	MacLellan et al. (2010)	Quebec	Empirical	Quantitative	IMGs			✓		
262.	MacPherson (2011)	Nova Scotia	Empirical	Qualitative	IMGs			✓		
263.	Magnus (2008)	Pan Canadian	Non Empirical		IMGs				✓	
264.	Mahamed, Gregory & Austin (2006)	Pan Canadian	Empirical	Quantitative	IEHPs			✓		
265.	Management Committee (2008)	Pan Canadian	Non Empirical		ITPs			✓		

Additional Table 2.
Thematic Coverage per Source

Source First Author (Year)		Location	Type of Paper	Methods	Profession	Themes				
						Pre-Immigration Activities & Programs	Early Arrival Activities & Programs	Professional Recertification	Alternative Paths to Integration	Workplace Integration
266.	Manitoba Regional Health Authority External Review Committee (2008)	Manitoba	Non Empirical		IMGs		✓			
267.	Marchildon & O'Fee (2008)	Saskatchewan	Non Empirical		IMGs					✓
268.	Masalmeh (2009)	Pan Canadian	Non Empirical		IMGs			✓		
269.	Matejicek (2008)	Ontario	Empirical	Qualitative	IMGs		✓			
270.	Mathews, Park & Rourke (2007)	Newfoundland & Labrador	Empirical	Qualitative	IMGs					✓
271.	Mathews, Edwards & Rourke (2008)	Newfoundland & Labrador	Empirical	Qualitative	IMGs					✓
272.	Mathews, Rourke & Park (2009)	Newfoundland & Labrador	Empirical	Quantitative	IMGs					✓
273.	Martin & Morgan (2005)	Pan Canadian	Non Empirical		IEMs			✓		
274.	Maudsley (2008)	Pan Canadian	Non Empirical		IMGs			✓	✓	
275.	Mayo & Mathews (2006)	Newfoundland & Labrador	Empirical	Qualitative	IMGs			✓		✓
276.	McDonald & Worswick (2010)	Pan Canadian	Empirical	Quantitative	IMGs					✓
277.	McDonald, Warman & Worswick (2010)	Pan Canadian	Empirical	Quantitative	IMGs					✓
278.	McGrath, Wong & Holewa (2011)	Pan Canadian	Empirical	Qualitative	IMGs			✓		
279.	McGuire & Murphy (2005)	Pan Canadian	Non Empirical		IENs			✓		
280.	McIntosh (2007)	Pan Canadian	Non Empirical		IEHPs	✓				

Additional Table 2.
Thematic Coverage per Source

Source<br>First Author (Year)		Location	Type of Paper	Methods	Profession	Themes				
						Pre-Immigration Activities & Programs	Early Arrival Activities & Programs	Professional Recertification	Alternative Paths to Integration	Workplace Integration
281.	McIntosh, Torgerson & Klassen (2007)	Pan Canadian	Empirical	Qualitative	IEHPs	✓				
282.	McKenna, Ganbesan & Soma (2007)	British Columbia	Empirical	Quantitative	IMGs					✓
283.	McMahon (2009)	Pan Canadian	Non Empirical		IMGs			✓		
284.	Med-Emg Inc. (2006)	Pan Canadian	Non Empirical		IENs					✓
285.	Medical Council of Canada (2011)	Pan Canadian	Non Empirical		IMGs			✓		
286.	Medical Council of Canada (2012)	Pan Canadian	Non Empirical		IMGs			✓		
287.	Meslay et al. (2008)	Quebec	Non Empirical		IENs		✓			✓
288.	Miller, Cooper & Eva (2010)	Pan Canadian	Empirical	Quantitative	PT			✓		
289.	Mills et al. (2008)	Pan Canadian	Non Empirical		IEHPs	✓				
290.	Ministry of Health and Long Term Care (2007)	Ontario	Non Empirical		IMGs		✓	✓		
291.	Minore, Pong & Ariss (2001)	Ontario	Non Empirical		IMGs					✓
292.	Mok et al. (2011)	Pan Canadian	Empirical	Quantitative	IMGs					✓
293.	Mont Royal University (2011)	Alberta	Report Empirical	Quantitative	IEHPs			✓		
294.	Muhammad Gadit (2008)	Pan Canadian	Empirical	Quantitative	IMGs	✓				
295.	Mullan (2005)	Pan Canadian	Empirical	Quantitative	IMGs	✓				
296.	Murphy (2008)	Alberta	Empirical	Qualitative	IENs	✓				

Additional Table 2.
Thematic Coverage per Source

Source<br>First Author (Year)		Location	Type of Paper	Methods	Profession	Themes				
						Pre-Immigration Activities & Programs	Early Arrival Activities & Programs	Professional Recertification	Alternative Paths to Integration	Workplace Integration
297.	Nasmith (2008)	Pan Canadian	Non Empirical		IMGs			✓		
298.	National Dental Assisting Examining Board (2011)	Pan Canadian	Non Empirical		ITDs			✓		
299.	National Dental Examining Board of Canada (2012)	Pan Canadian	Empirical	Quantitative	ITDs			✓		
300.	Neilsen (2010)	Pan Canadian	Empirical	Mixed Methods	IEHPs					✓
301.	Neiterman & Bourgeault (2012)	Pan Canadian	Empirical	Qualitative	IMGs		✓	✓		
302.	Nelson (2005)	British Columbia	Empirical	Qualitative	IENs	✓				
303.	Nelson et al. (2011)	Pan Canadian	Empirical	Qualitative	IENs					✓
304.	Newton, Pillay & Higginbottom (2012)	Pan Canadian	Non Empirical		IENs					✓
305.	Nguyen & Baptiste (2011)	Ontario	Non Empirical		IEOTs			✓		
306.	Nousiainen et al (2012)	Pan Canadian	Non Empirical		IMGs			✓		
307.	O'Brien-Pallas & Wang (2006)	Pan Canadian	Empirical	Quantitative	IENs					✓
308.	O'Meara (2004)	Pan Canadian	Non Empirical		IMGs			✓		
309.	Office of the Fairness Commissioner (2008)	Ontario	Empirical	Mixed Methods	IHEPs			✓		
310.	Office of the Fairness Commissioner (2009)	Ontario	Empirical	Qualitative	IEHPs			✓		
311.	Office of the Fairness Commissioner (2010a)	Ontario	Non Empirical		IEHPs	✓				
312.	Office of the Fairness Commissioner (2010b)	Ontario	Empirical	Mixed Methods	IEHPs			✓		

Additional Table 2.
Thematic Coverage per Source

Source First Author (Year)		Location	Type of Paper	Methods	Profession	Themes				
						Pre-Immigration Activities & Programs	Early Arrival Activities & Programs	Professional Recertification	Alternative Paths to Integration	Workplace Integration
313.	Official Languages Community Development Bureau (OLCDB) (2008)	Pan Canadian	Empirical	Qualitative	ITP			✓		
314.	Ogilvie & Long (2007)	Pan Canadian	Non Empirical		IENs			✓		
315.	Ogilvie et al (2007)	Pan Canadian	Non Empirical		IEHPs	✓				
316.	Ontario Ministry of Health and Long-Term Care (2005)	Pan Canadian	Non Empirical		IMGs		✓	✓		
317.	Ordre des infirmières et infirmiers du Québec (OIIQ) (2004)	Quebec	Non Empirical		IENs			✓		
318.	Ordre des infirmières et infirmiers du Québec (OIIQ) (2007)	Quebec	Non Empirical		IENs			✓		
319.	Ordre des infirmières et infirmiers du Québec (OIIQ) (2012)	Quebec	Non Empirical		IENs			✓		
320.	Osmond (2004)	Nova Scotia	Non Empirical		IEHPs					✓
321.	Pereira et al (2007)	Pan Canadian			IEHPs	✓				
322.	Peters (2011)	Ontario	Empirical	Qualitative	IMGs				✓	
323.	Phillip (2008)	Alberta	Non Empirical		IENs			✓		
324.	Pittman et al. (2012)	Alberta	Non Empirical		IENs	✓				
325.	Prince Edward Island-ANC (2011)	Prince Edward Island	Empirical	Mixed Methods	IEHPs		✓			

Additional Table 2.
Thematic Coverage per Source

	Source First Author (Year)	Location	Type of Paper	Methods	Profession	Themes: Pre-Immigration Activities & Programs	Early Arrival Activities & Programs	Professional Recertification	Alternative Paths to Integration	Workplace Integration
326.	Progress Centre Planning Institute (PCPI) (2011)	Pan Canadian	Empirical	Mixed Methods	IENs					✓
327.	Province of Nova Scotia (2012)	Nova Scotia	Non Empirical		IMGs					✓
328.	Province of Saskatchewan (2012)	Saskatchewan	Non Empirical		IMGs	✓				
329.	Pylypa (2011)	Pan Canadian	Empirical	Qualitative	IEHPs	✓				
330.	R.A. Malatest & Associates (2008)	Pan Canadian	Non Empirical		ITPs			✓		
331.	Rao (2012)	Pan Canadian	Empirical	Quantitative	IMGs			✓		
332.	Registered Nurses Association of Alberta (2008)	Alberta	Non Empirical		IENs			✓		
333.	Registered Nurses Association of Alberta (2012)	Alberta	Non Empirical		IENs			✓		
334.	Registered Nurses Association of Ontario (2008)	Ontario	Non Empirical		IENs	✓				
335.	Registered Nurses Association of Nova Scotia (2010)	Nova Scotia	Non Empirical		IENs	✓		✓		✓
336.	Ridewood (2010	Alberta	Non Empirical		IENs					✓
337.	Ronquillo (2007)	British Columbia	Empirical	Qualitative	IENs	✓				
338.	Ronquillo et al. (2011)	British Columbia	Empirical	Qualitative	IENs	✓				
339.	Ronquillo (2012)	British Columbia	Empirical	Qualitative	IENs	✓				
340.	Rosen (2001)	Pan Canadian	Non Empirical		IENs	✓				
341.	Rothman & Cusimano (2000)	Ontario	Empirical	Quantitative	IMGs				✓	

Additional Table 2.
Thematic Coverage per Source

Source First Author (Year)		Location	Type of Paper	Methods	Profession	Themes				
						Pre-Immigration Activities & Programs	Early Arrival Activities & Programs	Professional Recertification	Alternative Paths to Integration	Workplace Integration
342.	Rothman & Cusimano (2001)	Ontario	Empirical	Quantitative	IMGs		✓			
343.	Royal College of Physicians and Surgeons of Canada (2006)	Pan Canadian	Non Empirical		IMGs	✓				
344.	Royal College of Physicians and Surgeons of Canada (2009)	Pan Canadian	Non Empirical		IMGs			✓		
345.	Royal College of Physicians and Surgeons of Canada (2012)	Pan Canadian	Non Empirical		IMGs					✓
346.	Runnels, Labonte & Packer (2011)	Pan Canadian	Empirical	Qualitative	IEHPs	✓				
347.	Salma (2009)	Alberta	Empirical	Qualitative	IENs					✓
348.	Salma, Kegadoren & Ogilvie (2012)	Alberta	Empirical	Qualitative	IENs					✓
349.	Santa Mina et al. (2011)	Ontario	Empirical	Mixed Methods	IENs			✓		
350.	Saskatchewan Ministry of Health (2005)	Saskatchewan	Non Empirical		IMGs	✓		✓		
351.	Saskatchewan Ministry of Health (2010)	Saskatchewan	Empirical	Quantitative	IMGs	✓				
352.	Saskatchewan Registered Nurses Association (2008a)	Saskatchewan	Empirical	Qualitative	IENs	✓				
353.	Saskatchewan Registered Nurses Association (2008b)	Saskatchewan	Non Empirical		IENs	✓				
354.	Saunders (2008)	Nova Scotia	Empirical	Qualitative	IEHPs			✓		✓

Additional Table 2.
Thematic Coverage per Source

	Source<br>First Author (Year)	Location	Type of Paper	Methods	Profession	Themes: Pre-Immigration Activities & Programs	Themes: Early Arrival Activities & Programs	Themes: Professional Recertification	Themes: Alternative Paths to Integration	Themes: Workplace Integration
355.	Scarrow (2008)	Ontario	Non Empirical		IENs	✓				
356.	Schwartz (2012)	Pan Canadian	Non Empirical		IEHPs			✓		✓
357.	Sharieff & Zakus (2006)	Ontario	Empirical	Mixed Methods	IMGs			✓		
358.	Shuchman (2008)	Pan Canadian	Non Empirical		IMGs	✓				
359.	Singh & Sochan (2010)	Ontario	Non Empirical		IENs			✓		
360.	Slade (2008)	Pan Canadian	Empirical	Quantitative	IMGs	✓				
361.	Sochan & Singh (2007)	Ontario	Empirical	Qualitative	IENs			✓		
362.	Society of Rural Physicians of Canada (2002)	Pan Canadian	Non Empirical		IEHPs			✓		
363.	Sockalingam et al. (2012)	Pan Canadian	Empirical	Quantitative	IMGs			✓		
364.	Spurgeon (2000)	Pan Canadian	Non Empirical		IMGs			✓		
365.	Standing Committee on Human Resources Skills and Social Development and the Status of Persons with Disabilities, (2012)	Pan Canadian	Non Empirical		IEHPs			✓		
366.	Standing Senate Committee on Social Affairs, Science and Technology (2012)	Pan Canadian	Non Empirical		IEHPs			✓		
367.	Stenerson, Davis & Labash (2012)	Saskatchewan	Non Empirical		IMGs				✓	
368.	Szafran et al. (2005)	Pan Canadian	Empirical	Quantitative	IMGs			✓		

Additional Table 2.
Thematic Coverage per Source

Source First Author (Year)		Location	Type of Paper	Methods	Profession	Themes				
						Pre-Immigration Activities & Programs	Early Arrival Activities & Programs	Professional Recertification	Alternative Paths to Integration	Workplace Integration
369.	Taylor, Foster & Cambre (2012)	Alberta	Non Empirical		IENs					✓
370.	The Maytree Foundation (2001)	Pan Canadian	Non Empirical		IMGs					✓
371.	The University of British Columbia (2012)	British Columbia	Empirical	Quantitative	ITDs	✓				
372.	Thind et al. (2007)	Ontario	Empirical	Quantitative	IMGs					✓
373.	Thind et al (2008)	Ontario	Empirical	Quantitative	IMGs					✓
374.	Tilley (2007)	Pan Canadian	Non Empirical		IENs				✓	
375.	Toguri, Jong, Roger (2012)	Pan Canadian	Empirical	Quantitative	IENs			✓		✓
376.	Tomson & Cohl (2011)	Ontario	Empirical	Mixed Methods	IMGs			✓		
377.	Tregunno et al (2007)	Ontario	Non Empirical		IENs			✓		
378.	Tregunno et al. (2009)	Ontario	Non Empirical		IENs					✓
379.	Truscott (2008)	Pan Canadian	Empirical	Quantitative	IMGs			✓		
380.	Turner (2009)	Saskatchewan	Non Empirical		IENs			✓		
381.	Turrittin et al. (2002)	Pan Canadian	Empirical	Qualitative	IENs					✓
382.	Urowitz (2008)	Ontario	Non Empirical		IMGs			✓		
383.	Vallevand & Violato (2012)	Pan Canadian	Empirical	Quantitative	IMGs			✓		
384.	Van Iterson (2010)	Pan Canadian	Non Empirical		IEOTs			✓		

Additional Table 2.
Thematic Coverage per Source

	Source<br>First Author (Year)	Location	Type of Paper	Methods	Profession	Themes				
						Pre-Immigration Activities & Programs	Early Arrival Activities & Programs	Professional Recertification	Alternative Paths to Integration	Workplace Integration
385.	Van Iterson (2011)	Pan Canadian	Non Empirical		IEOTs				✓	
386.	Vandersloot (2009)	Ontario	Empirical	Qualitative	IEMs			✓		
387.	Vardy, Ryan & Audas (2008)	Newfoundland & Labrador	Empirical	Quantitative	IMGs					✓
388.	von Zweck (2006)	Pan Canadian	Empirical	Quantitative	OTs			✓		
389.	Waddell (2006)	Pan Canadian	Non Empirical		IMGs			✓		
390.	Walsh et al (2011)	Pan Canadian	Empirical	Qualitative	IMGs			✓		
391.	Ward (2009)	Pan Canadian	Empirical	Quantitative				✓		
392.	Watanabe (2008)	Pan Canadian	Empirical	Quantitative	IMGs					✓
393.	Watt et al. (2010)	Alberta	Non Empirical		IMGs			✓		
394.	Watt, Violato & Lake (2012)	Alberta	Non Empirical		IMGs			✓		
395.	Watt et al. (2003)	Alberta	Non Empirical		IMGs			✓		
396.	Watt et al. (2010)	Alberta	Non Empirical		IMGs				✓	
397.	Watts, Davies & Metcalfe (2011)	Pan Canadian	Empirical	Quantitative	IMGs			✓		
398.	Weerasekera (2012)	Pan Canadian	Non Empirical		IMGs			✓		
399.	Western Alliance for Assessment of International Physicians [WAAIP] (2006)	Pan Canadian	Non Empirical		IMGs		✓	✓	✓	

Additional Table 2.
Thematic Coverage per Source

Source First Author (Year)		Location	Type of Paper	Methods	Profession	Themes				
						Pre-Immigration Activities & Programs	Early Arrival Activities & Programs	Professional Recertification	Alternative Paths to Integration	Workplace Integration
400.	Western and Northern Health Human Resources and Planning Forum (2006a)	Western Provinces & Territories	Non Empirical		IEHPs		✓	✓		
401.	Western and Northern Health Human Resources and Planning Forum (2006b)	Western Provinces & Territories	Non Empirical		IMGs		✓	✓		✓
402.	Wharry (2002)	Pan Canadian	Non Empirical		IMGs	✓				
403.	Wong & Lohfeld (2008)	Ontario	Empirical	Qualitative	IMGs			✓		
404.	Xu & Zhang (2005)	Pan Canadian	Non Empirical		IENs	✓				
405.	Yan (2006)	Pan Canadian	Non Empirical		IENs	✓				
406.	Zaman (2006)	Pan Canadian	Empirical	Qualitative	IEHPs			✓	✓	✓
407.	Zulla et al. (2008)	Pan Canadian	Empirical	Quantitative	IMGs				✓	

N.B. IEDs= internationally educated dieticians; IEHPs= internationally educated health professionals; IEMLTs=internationally educated medical laboratory technologists; IEMs= internationally educated midwives, IENs= internationally educated nurses; IEOTs= internationally educated occupational therapists, IERTs= internationally educated respiratory technologists; ITDs=internationally trained dentists; IMGs=international medical graduates; ITPs= internationally trained pharmacists; ITMRTs =internationally trained medical radiation technologists; IEPs=internationally educated physiotherapists.
